# Supplementary material for: Early-phase impact of obesity-associated stress on murine vascular smooth muscle cells depends on EGFR and sex
Source: Commun Biol. 2025 Dec 22;8:1834. doi: 10.1038/s42003-025-09416-7 (PMC12749158; doi:10.1038/s42003-025-09416-7)
Supplement: Supplementary file 1 — Supplementary material [file 42003_2025_9416_MOESM1_ESM.pdf]

Supplementary material for

Early-phase impact of obesity-associated stress on murine vascular smooth muscle cells depends on EGFR and sex

Authors:
V.D.*, S.R.*, N.N.-A., M.K., S.M., G.S., B.S.#, M.G.#

* equal contribution
# equal contribution

Affiliation:
Julius-Bernstein-Institute of Physiology, Martin Luther University Halle-Wittenberg, Halle, Germany

Address of correspondence:
Prof. Dr. Michael Gekle, MD
Julius-Bernstein-Institute of Physiology
Martin-Luther-University Halle-Wittenberg
Magdeburger Strasse 6
06112 Halle (Saale)
Germany
E-Mail: michael.gekle@medizin.uni-halle.de
ORCID ID: 0000-0002-1581-8767

© Michael Gekle | Julius-Bernstein-Institut für Physiologie | Martin-Luther-Universität Halle-Wittenberg

Early-phase impact of obesity-associated stress on murine vascular smooth muscle cells depends on EGFR and sex

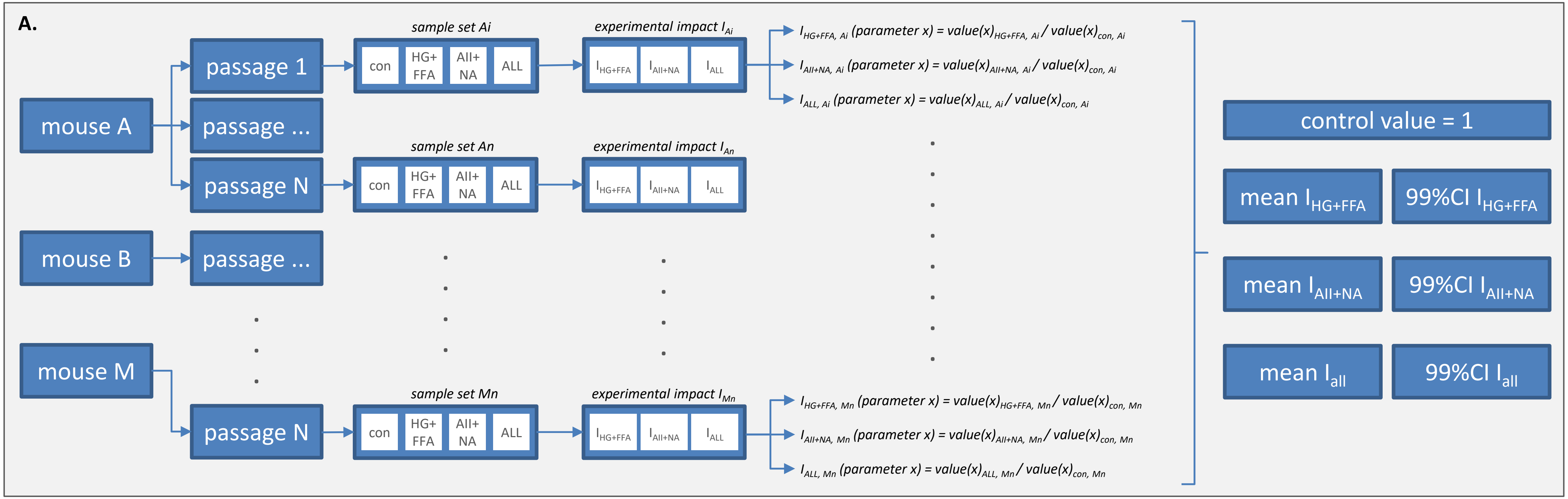

B.

animal & cell culture inventory			
experiments	cell type	number of mice used	independent sets (animals × passages)
RNASeq (male animals)	EC	9	10
	WT VSMC	4	8
	KO VSMC	4	8
validadtion experiments	EC	12	>19
	WT VSMC	11	>23
	KO VSMC	8	>16

SF1. A) Our experimental design provided strictly connected sets of samples for control, metabolic, humoral or combined stressors originating from the same animal, at the same passage, treated at exactly the same time. This design allowed us to calculate the relative effects of the stressors in a paired way (stressor effect = $value_{stressor}/value_{control}$). Subsequently, we could the 95% or 99% confidence intervals were used to test for significance at the level of $\alpha < 0.05$ or 0.01). B) Number of animals and independent sets.

© Michael Gekle | Julius-Bernstein-Institut für Physiologie | Martin-Luther-Universität Halle-Wittenberg

Early-phase impact of obesity-associated stress on murine vascular smooth muscle cells depends on EGFR and sex

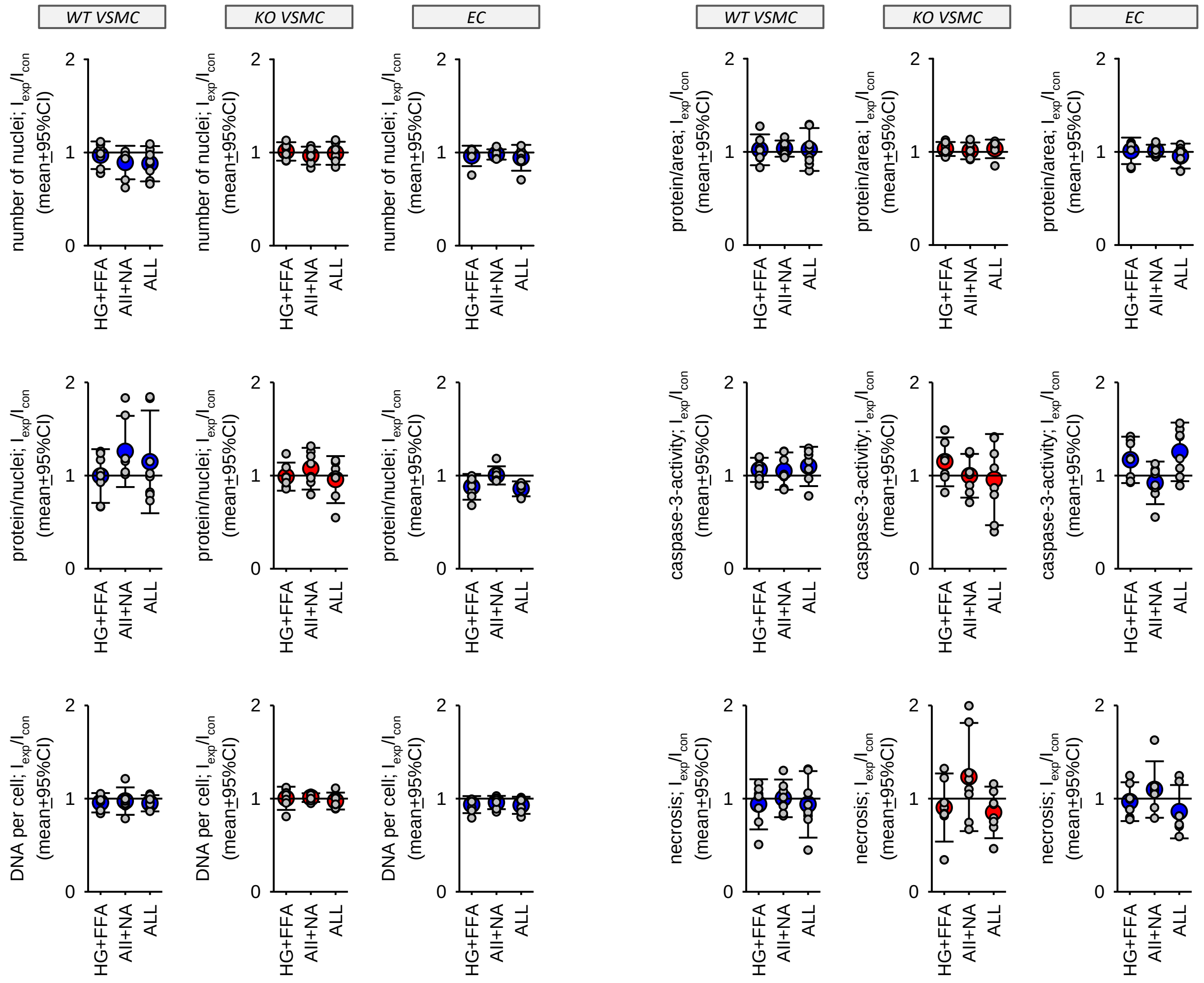

SF2. Effect of the stressors on the number of nuclei (i.e. number of cells), nuclear DNA content, cellular protein, the apoptosis marker caspase-3-activity and necrosis (determined by trypan blue exclusion). N = 8 plates with up to 6 wells for each condition and each cell type.

WT VSMC	Con	HG+FFA	AII+NA	ALL
∅ rel. sd	0.19	0.20	0.19	0.17
	FC ∉ [0.64; 1.56] ➡ $\lvert \log_2 FC \rvert > 0.64$			

KO VSMC	Con	HG+FFA	AII+NA	ALL
∅ rel. sd	0.263	0.208	0.208	0.186
	FC ∉ [0.61; 1.63]			

EC	Con	HG+FFA	AII+NA	ALL
∅ rel. sd	0.35	0.32	0.34	0.33
	FC ∉ [0.50; 2.00]			

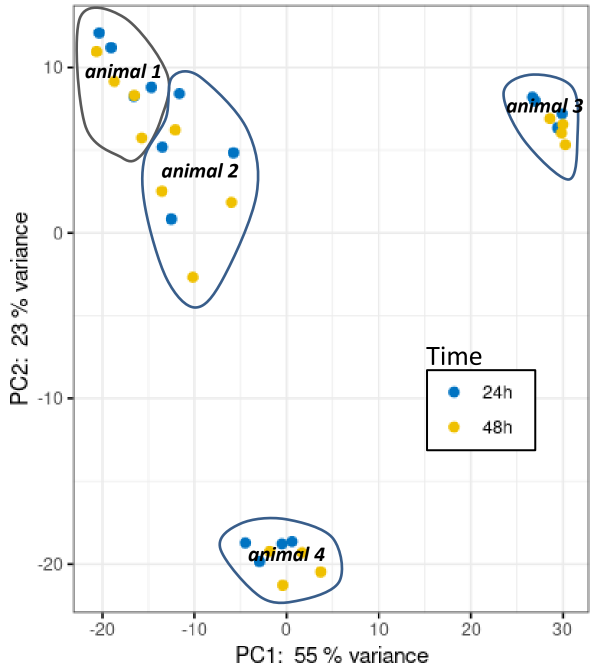

SF3. The tables show the average relative standard deviation (∅ rel. sd = Σsd FPM_{RNAi}/number of RNAs; for i=1 to n) of FPM for all protein coding RNAs and lncRNAs. Fold change threshold were derived from the average relative sd x 3. Upper FC threshold = 1 + (average relative sd x 3). Lower FC threshold = 1 / upper threshold. The $\log_2$FC thresholds were then derived from the FC threshold values. The right panel shows as an example the PC analysis for WT VSMC control samples from 4 animals, harvested after 24 or 48 h. The data clearly show the strong clustering determined by the donor (animal).

Cell type		WT VSMC											
		HG+FFA				AII+NA				ALL			
Process	GOI	99%CI upper limit	99%CI lower limit	mean		99%CI upper limit	99%CI lower limit	mean		99%CI upper limit	99%CI lower limit	mean	
adrenergic signaling	ADRA1B	1,89	1,14	1,52	1	2,24	1,04	1,64	1	2,61	1,60	2,10	1
adrenergic signaling	ADRB3	1,83	1,10	1,47	1	1,81	0,89	1,35	0	1,97	1,15	1,56	1
AII-signaling	AGTR1A	1,27	0,77	1,02	0	2,08	1,26	1,67	1	2,26	1,14	1,70	1
AII-signaling	AGTR1B	1,19	0,79	0,99	0	3,14	1,68	2,41	1	2,97	1,08	2,03	1
AII-signaling	AGTR2	1,67	0,51	1,09	0	0,75	0,14	0,45	-1	0,48	-0,02	0,23	-1
differentiation	CD34	0,65	0,40	0,52	-1	0,87	0,58	0,72	-1	0,46	0,28	0,37	-1
differentiation	CDH5	1,33	0,14	0,74	0	1,17	0,12	0,65	0	0,45	-0,07	0,19	-1
differentiation	MYL12B	1,16	0,98	1,07	0	1,87	1,24	1,55	1	2,01	1,25	1,63	1
differentiation	MYLIP	0,79	0,47	0,63	-1	1,11	0,93	1,02	0	0,69	0,48	0,59	-1
differentiation	MYLK	1,58	0,89	1,24	0	2,63	1,52	2,07	1	3,25	1,39	2,32	1
differentiation	TAGLN	1,45	0,74	1,10	0	2,51	1,21	1,86	1	2,39	1,27	1,83	1
fibrosis	ANGPTL4	4,49	1,46	2,98	1	1,19	0,87	1,03	0	4,39	1,42	2,90	1
fibrosis	COL8A1	1,48	1,12	1,30	1	1,87	0,95	1,41	0	2,23	1,20	1,72	1
fibrosis	COL8A2	0,80	0,44	0,62	-1	1,12	0,61	0,87	0	0,87	0,31	0,59	-1
fibrosis	LY6A	0,87	0,55	0,71	-1	1,00	0,74	0,87	0	0,80	0,43	0,61	-1
inflammation	CCL7	1,31	0,45	0,88	0	2,19	0,58	1,39	0	0,92	0,33	0,62	-1
inflammation	FLT1	1,19	0,85	1,02	0	2,39	1,35	1,87	1	2,49	1,19	1,84	1
inflammation	LGALS3	0,73	0,51	0,62	-1	1,23	0,79	1,01	0	0,73	0,52	0,63	-1

Cell type		KO VSMC											
		HG+FFA				AII+NA				ALL			
Process	GOI	99%CI upper limit	99%CI lower limit	mean		99%CI upper limit	99%CI lower limit	mean		99%CI upper limit	99%CI lower limit	mean	
adrenergic signaling	ADRA1B	1,54	0,94	1,24	0	1,56	0,87	1,22	0	1,51	0,97	1,24	0
adrenergic signaling	ADRB3	1,75	1,00	1,38	0	1,07	0,69	0,88	0	1,71	0,73	1,22	0
AII-signaling	AGTR1A	2,24	0,34	1,29	0	3,36	0,51	1,93	0	2,89	0,11	1,50	0
AII-signaling	AGTR1B	1,43	0,49	0,96	0	2,07	1,21	1,64	1	1,10	0,51	0,81	0
AII-signaling	AGTR2	1,46	0,30	0,88	0	0,89	0,14	0,52	-1	1,45	0,05	0,75	0
differentiation	CD34	0,97	0,66	0,82	-1	0,96	0,78	0,87	-1	1,03	0,70	0,86	0
differentiation	CDH5	1,79	0,21	1,00	0	3,91	0,09	2,00	0	#DIV/0!	#DIV/0!		
differentiation	MYL12B	1,11	0,86	0,98	0	1,27	0,92	1,10	0	1,11	0,80	0,96	0
differentiation	MYLIP	0,72	0,50	0,61	-1	1,17	1,01	1,09	1	0,69	0,44	0,57	-1
differentiation	MYLK	1,95	0,66	1,30	0	2,34	0,72	1,53	0	1,65	0,78	1,21	0
differentiation	TAGLN	1,13	0,79	0,96	0	1,30	0,79	1,04	0	1,29	0,65	0,97	0
fibrosis	ANGPTL4	8,61	2,51	5,56	1	0,89	0,59	0,74	-1	10,56	3,11	6,83	1
fibrosis	COL8A1	1,44	0,90	1,17	0	1,37	1,01	1,19	1	1,67	0,94	1,30	0
fibrosis	COL8A2	0,68	0,41	0,55	-1	1,21	0,70	0,95	0	0,48	0,30	0,39	-1
fibrosis	LY6A	1,03	0,73	0,88	0	0,93	0,71	0,82	-1	0,97	0,65	0,81	-1
inflammation	CCL7	1,86	0,70	1,28	0	0,92	0,68	0,80	-1	0,99	0,66	0,82	-1
inflammation	FLT1	1,02	0,54	0,78	0	1,78	1,17	1,47	1	1,49	0,58	1,03	0
inflammation	LGALS3	0,77	0,48	0,63	-1	1,08	0,80	0,94	0	0,84	0,38	0,61	-1

Cell type		EC											
		HG+FFA				AII+NA				ALL			
Process	GOI	99%CI upper limit	99%CI lower limit	mean		99%CI upper limit	99%CI lower limit	mean		99%CI upper limit	99%CI lower limit	mean	
adrenergic signaling	ADRA1B	2,00	0,53	1,26	0	1,11	0,27	0,69	0	1,17	0,45	0,81	0
adrenergic signaling	ADRB3	1,45	0,30	0,88	0	1,42	0,15	0,79	0	1,99	0,29	1,14	0
AII-signaling	AGTR1A	1,26	0,07	0,67	0	1,26	0,07	0,67	0	2,32	0,13	1,22	0
AII-signaling	AGTR1B	#NV	#NV			#NV	#NV			#NV	#NV		
AII-signaling	AGTR2	#NV	#NV			#NV	#NV			#NV	#NV		
differentiation	CD34	1,64	0,38	1,01	0	1,25	0,53	0,89	0	1,22	0,54	0,88	0
differentiation	CDH5	1,76	0,63	1,19	0	1,25	0,49	0,87	0	1,38	0,75	1,07	0
differentiation	MYL12B	1,67	0,53	1,10	0	1,20	0,49	0,84	0	1,27	0,61	0,94	0
differentiation	MYLIP	0,71	0,20	0,45	-1	1,21	0,52	0,87	0	0,62	0,30	0,46	-1
differentiation	MYLK	1,57	0,52	1,05	0	1,37	0,51	0,94	0	1,43	0,58	1,00	0
differentiation	TAGLN	4,53	0,34	2,44	0	1,23	0,45	0,84	0	1,99	0,69	1,34	0
fibrosis	ANGPTL4	23,56	3,44	13,50	1	1,29	0,43	0,86	0	19,28	4,34	11,81	1
fibrosis	COL8A1	1,52	0,69	1,11	0	1,35	0,59	0,97	0	1,37	0,75	1,06	0
fibrosis	COL8A2	1,10	0,21	0,65	0	1,69	0,49	1,09	0	0,88	0,12	0,50	-1
fibrosis	LY6A	1,08	0,25	0,66	0	1,37	0,48	0,92	0	1,17	0,52	0,85	0
inflammation	CCL7	1,57	0,49	1,03	0	1,62	0,55	1,09	0	1,59	0,68	1,13	0
inflammation	FLT1	1,31	0,40	0,86	0	1,27	0,51	0,89	0	0,97	0,54	0,76	-1
inflammation	LGALS3	1,55	0,38	0,97	0	1,11	0,37	0,74	0	1,17	0,53	0,85	0

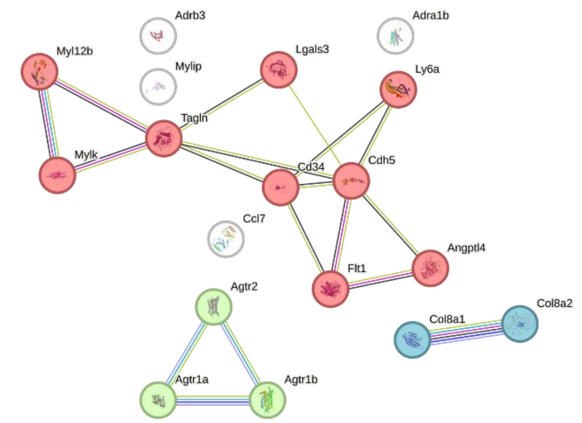

#clustering method	cluster number	cluster color	gene count	primary description	protein names
kmeans	1	Red	9	Myosin II complex, and Mesenchyme migration	Flt1, Cdh5, Tagln, Myl12b, Mylk, Lgals3, Cd34, Ly6a, Angptl4
kmeans	2	Green	3	Maintenance of blood vessel diameter homeostasis by renin-angiotensin	Agtr2, Agtr1a, Agtr1b

SF4. DEG analysis by a directed approach. Investigation of potential changes in RNA abundance for genes known to be typically affected during pathological vascular alterations (biomarker genes). The upper and lower limits of the confidence intervals for the respective stressors impact ($FPM_{stressor}/FPM_{control}$) are shown. Exclusion of the value 1 from the confidence intervals was used as test criterium. For each cell type and each condition 4 independent biological replicates were included in the differential expression analysis. The lower panel show the result from STRING analysis using DEG identified for WT VSMC after exposure to all stressors.

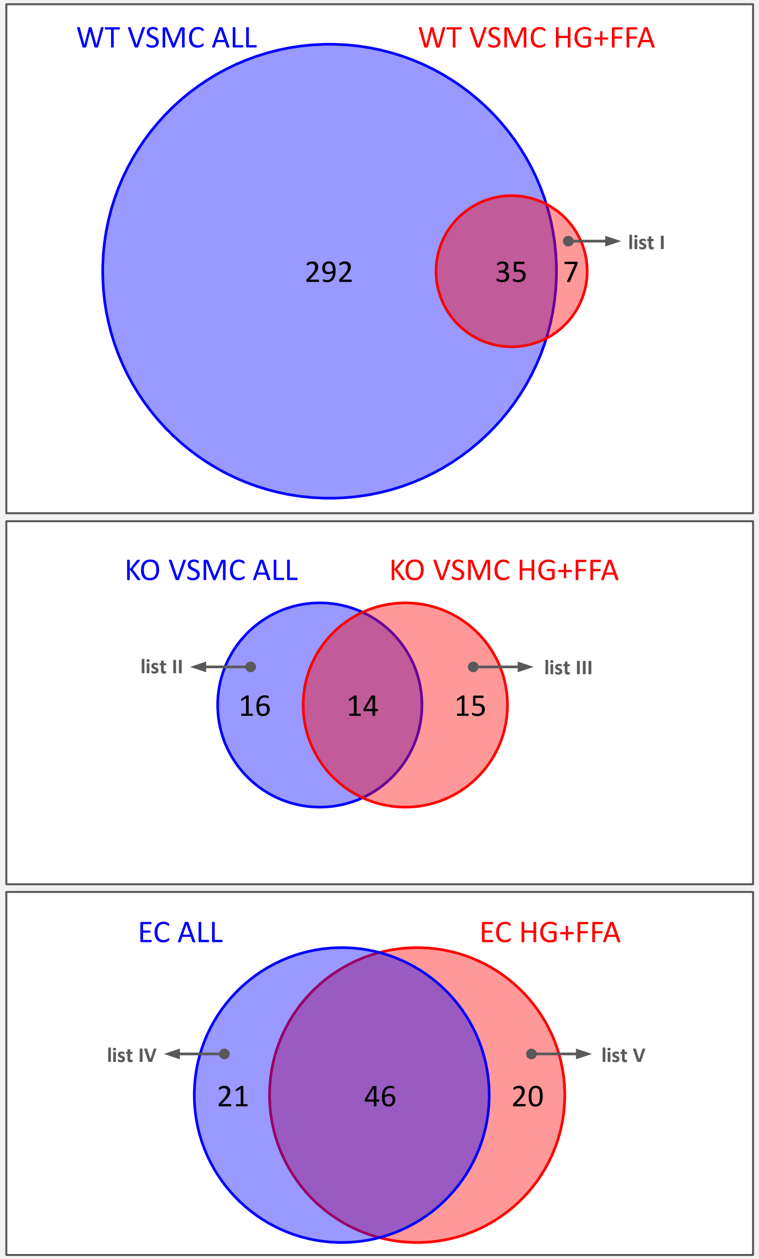

list I	HG+FFA		All+NA		ALL	
95% CI	UL	LL	UL	LL	UL	LL
Ckap2	1,75	1,33	1,20	0,93	1,88	1,27
Cldn15	2,42	1,71	1,21	0,66	2,49	1,13
Gm14403	3,47	1,37	1,40	0,67	2,12	0,96
Hsd17b7	1,84	1,37	1,40	1,02	1,91	1,31
Lgals3	0,73	0,51	1,23	0,79	0,73	0,52
Pals2	1,95	1,15	1,00	0,83	1,70	1,06
Prss35	2,55	0,96	1,04	0,36	1,98	0,80

list II	HG+FFA		All+NA		ALL	
95% CI	UL	LL	UL	LL	UL	LL
Abat	0,86	0,55	1,30	0,99	0,75	0,44
Aldh1a1	1,67	1,32	1,17	0,92	1,92	1,39
Angptl4	9,02	1,20	0,95	0,51	9,91	2,36
Ankrd1	1,89	0,91	1,37	1,19	2,44	1,28
Cbs	0,95	0,48	1,02	0,84	0,61	0,21
Dlk1	0,84	0,29	1,09	0,46	0,60	0,17
Dusp27	5,47	1,33	2,37	1,03	7,00	1,40
Gfra1	0,86	0,54	1,09	0,93	0,72	0,42
Hmox1	1,58	1,19	1,20	0,85	1,87	1,80
Hsd11b1	0,97	0,53	0,93	0,64	0,68	0,49
Klf15	0,74	0,60	1,08	0,88	0,59	0,49
Pde1b	2,09	0,98	1,41	0,87	2,23	1,52
Plekha4	0,99	0,57	0,98	0,67	0,75	0,36
Pparg	2,61	1,06	1,54	0,79	2,69	1,75
Ppp1r3c	0,76	0,35	0,99	0,84	0,67	0,27
Siglecg	1,67	0,87	1,61	0,77	2,09	1,98

list III	HG+FFA		All+NA		ALL	
95% CI	UL	LL	UL	LL	UL	LL
Arrdc4	0,75	0,37	1,14	0,93	0,58	0,55
Ccdc148	0,74	0,09	1,27	0,90	0,67	0,44
Cdon	2,45	1,01	1,55	0,89	1,88	1,37
Gabre	1,99	1,53	1,16	0,55	1,82	1,31
Gm8113	2,79	1,46	1,82	0,77	2,07	1,62
Heg1	2,97	1,15	1,54	0,92	2,08	1,83
Lgals3	0,72	0,46	1,10	0,69	0,79	0,47
Lgals4	2,48	1,27	1,16	0,80	2,43	1,19
Lypd1	0,84	0,40	1,21	0,79	0,87	0,28
Mbd1	2,87	1,25	1,16	0,98	2,84	0,90
S1pr3	1,92	1,40	1,07	0,82	2,31	1,47
Scarb2	2,28	1,18	1,20	0,85	2,52	1,13
Slc22a5	2,13	1,24	1,01	0,97	1,82	1,37
Slc25a42	2,29	1,41	1,17	0,92	1,96	1,48
Tnfrsf1b	1,91	1,40	1,10	0,85	1,59	1,36

list IV	HG+FFA		All+NA		ALL	
95% CI	UL	LL	UL	LL	UL	LL
Baz1a	2,21	1,03	1,57	0,59	2,17	1,09
Dbi	2,10	1,02	1,35	0,57	2,13	1,09
Dhx58	0,94	0,44	1,44	0,55	0,93	0,41
Garin5a	1,10	0,50	1,20	0,39	0,77	0,37
Gm26532	0,93	0,53	1,18	0,45	0,91	0,37
Gramd1b	0,82	0,43	1,46	0,60	0,74	0,35
Hmox1	2,47	1,00	1,34	0,53	2,17	1,14
Irf7	1,09	0,49	1,40	0,45	0,88	0,42
Isg15	0,96	0,38	1,41	0,46	0,87	0,23
Lgr6	1,20	0,08	1,87	0,14	1,15	0,04
Mdga1	0,94	0,43	1,21	0,45	0,95	0,36
Ndrg1	0,88	0,48	1,18	0,51	0,86	0,47
Neu3	2,05	1,13	1,49	0,62	2,10	1,12
Oasl2	0,94	0,48	1,47	0,47	0,96	0,46
Olfml2a	0,93	0,31	1,41	0,44	0,93	0,27
Rnft2	1,11	0,47	1,47	0,47	1,01	0,33
Rrm2	2,98	0,76	2,15	0,31	3,38	0,68
S100a7a	0,97	0,44	1,13	0,35	0,95	0,41
Slc16a6	0,98	0,49	0,92	0,34	0,73	0,39
Snhg20	1,01	0,55	1,12	0,45	0,89	0,42
X.1192	3,01	1,06	1,47	0,57	3,17	1,40

list V	HG+FFA		All+NA		ALL	
95% CI	UL	LL	UL	LL	UL	LL
Ccdc74a	0,81	0,27	1,20	0,41	0,89	0,49
Cfap126	1,04	0,38	1,46	0,46	1,23	0,34
Crip1	0,82	0,31	1,26	0,50	0,91	0,44
Cspg4	0,81	0,20	1,07	0,25	1,15	0,20
Cxcl15	0,66	0,11	1,43	0,33	0,98	0,04
Dgkh	2,63	1,21	1,78	0,66	2,11	0,98
Dkk3	0,97	0,40	1,16	0,42	1,02	0,43
Dnm1	1,20	0,32	1,18	0,42	1,03	0,51
Egr2	0,76	0,16	1,48	0,50	1,01	0,29
Gm43672	0,80	0,41	1,21	0,39	0,89	0,43
Gm8113	4,20	0,69	2,01	0,62	3,61	0,54
Heg1	2,08	1,09	1,40	0,57	1,89	1,02
Hmgn2.ps	0,92	0,41	1,87	0,40	1,43	0,60
Lgals4	2,61	1,18	1,36	0,41	2,08	1,07
Mpst	0,84	0,42	1,00	0,43	0,89	0,50
Psmb9	0,85	0,38	0,98	0,39	1,00	0,49
Resf1	2,19	1,12	1,48	0,62	2,19	0,98
Serpinb9b	2,35	0,94	1,50	0,58	2,21	0,89
Sugct	0,85	0,40	1,13	0,35	0,97	0,43
Zfp946	3,15	0,98	1,48	0,56	2,13	0,97

UL = upper limit of 95% confidence interval for $Impact_{stressor} = FPM_{exp}/FPM_{con}$
LL = lower limit of 95% confidence interval for $Impact_{stressor} = FPM_{exp}/FPM_{con}$
When UL and LL > 1 or UL and LL < 1 = significant $Impact_{stressor}$

SF5A. Analysis of different overlaps. Expression of genes were checked according to the relative effects of the stressors determined in a paired way (see methods).

A) Comparison of the metabolic and ALL stressor effect in WT VSMC, KO VSMC and EC. For WT VSMC, KO VSMC and each condition 4 independent biological replicates were included in the differential expression analysis. For EC and each condition 10 independent biological replicates were included in the differential expression analysis. UL = upper limit of 95% confidence interval for $Impact_{stressor} = FPM_{exp}/FPM_{con}$. LL = lower limit of 95% confidence interval for $Impact_{stressor} = FPM_{exp}/FPM_{con}$
When UL and LL > 1 or UL and LL < 1 = significant $Impact_{stressor}$

HG+FFA	WT VSMC		KO VSMC	
95% CI	UL	LL	UL	LL
AI838599	4,32	1,40	3,91	0,43
Aldh1a3	0,55	0,32	0,67	0,22
Angptl7	0,67	0,42	0,91	0,34
Apod	0,55	0,24	0,71	0,27
Ccnb1	2,53	1,19	2,23	1,10
Cd34	0,65	0,40	0,97	0,66
Ckap2	1,75	1,33	1,82	1,12
Cldn15	2,42	1,71	2,31	0,98
Col5a3	0,85	0,49	0,72	0,55
Dhrs3	2,88	1,60	2,08	1,30
Epha7	1,98	1,42	1,70	1,23
Gm14403	3,47	1,37	1,93	0,71
Hsd17b7	1,84	1,37	2,40	0,61
Ifi27l2a	1,17	0,26	1,73	0,70
Kif11	2,47	1,03	2,64	0,56
Klf2	0,66	0,44	0,78	0,47
Lipe	2,21	1,44	2,75	1,55
Mki67	3,23	1,06	3,12	0,87
Ncaph	1,93	1,25	1,80	1,08
Pals2	1,95	1,15	1,59	1,01
Pcdh11x	3,70	1,72	2,77	0,23
Pdgfd	1,78	1,45	1,57	1,08
Pfkfb3	2,18	1,21	2,35	0,69
Prl2c2	3,70	0,58	7,93	1,36
Prss35	2,55	0,96	2,03	0,22
Rrs1	1,71	1,24	1,46	1,02
Spire2	0,88	0,54	0,93	0,59
Top2a	2,19	1,49	3,65	0,58
Zfp970	4,18	2,02	3,03	1,14

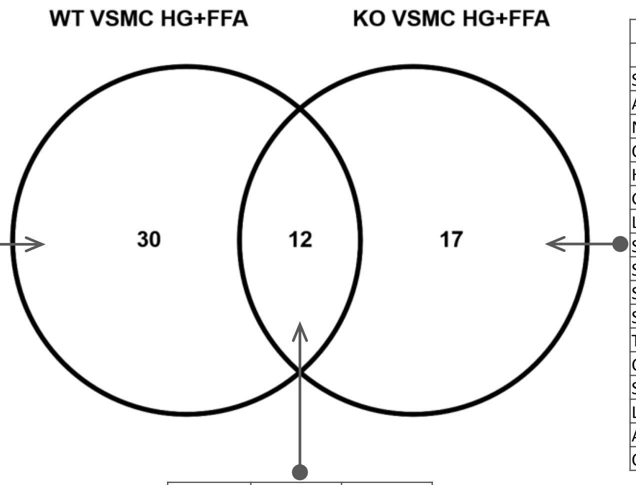

HG+FFA	WT VSMC		KO VSMC	
95% CI	UL	LL	UL	LL
Stbd1	5,31	1,70	8,85	2,31
Adcy1	2,72	1,43	2,61	1,21
Npy1r	2,13	1,03	3,13	1,59
Cpt1a	1,72	1,13	2,38	1,27
Heg1	1,82	1,20	2,45	1,20
Gm8113	2,40	1,16	2,76	1,59
Lgals4	1,70	1,24	2,36	1,40
Slc25a42	1,66	1,07	1,94	1,22
S1pr3	1,73	1,27	2,32	1,43
Scarb2	1,78	1,24	2,14	1,38
Slc22a5	1,47	1,04	1,86	1,24
Tnfrsf1b	1,73	1,21	1,69	1,29
Cdon	1,40	0,97	1,91	1,01
Scd2	0,95	0,60	0,69	0,51
Lypd1	0,93	0,55	0,71	0,48
Arrdc4	0,98	0,61	0,91	0,48
Ccdc148	1,41	-0,08	0,90	0,34

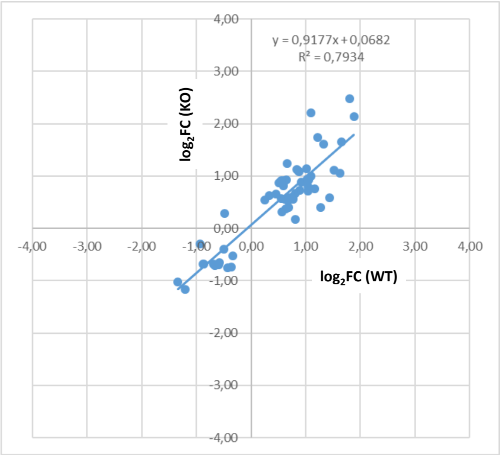

HG+FFA	WT VSMC		KO VSMC	
95% CI	UL	LL	UL	LL
Ech1	1,83	1,29	2,23	1,57
Gabra3	6,03	1,33	6,84	1,97
Gabre	2,55	1,51	2,22	1,62
Gria4	2,95	1,68	4,40	2,27
Hmgcs2	3,49	1,55	3,91	2,17
Klhl13	2,45	1,34	2,27	1,42
Lgals3	0,73	0,51	0,77	0,48
Mbd1	2,59	1,54	2,29	1,19
Mylip	0,79	0,47	0,72	0,50
Pdk4	4,34	1,94	4,08	2,22
Slc43a3	2,48	1,57	2,81	1,60
Tgm2	2,05	1,36	1,76	1,24

SF5B. Comparison of the metabolic stressor effect in WT VSMC and KO VSMC. For each cell type and each condition 4 independent biological replicates were included in the differential expression analysis.
UL = upper limit of 95% confidence interval for $Impact_{stressor} = FPM_{exp}/FPM_{con}$. LL = lower limit of 95% confidence interval for $Impact_{stressor} = FPM_{exp}/FPM_{con}$
When UL and LL > 1 or UL and LL < 1 = significant $Impact_{stressor}$

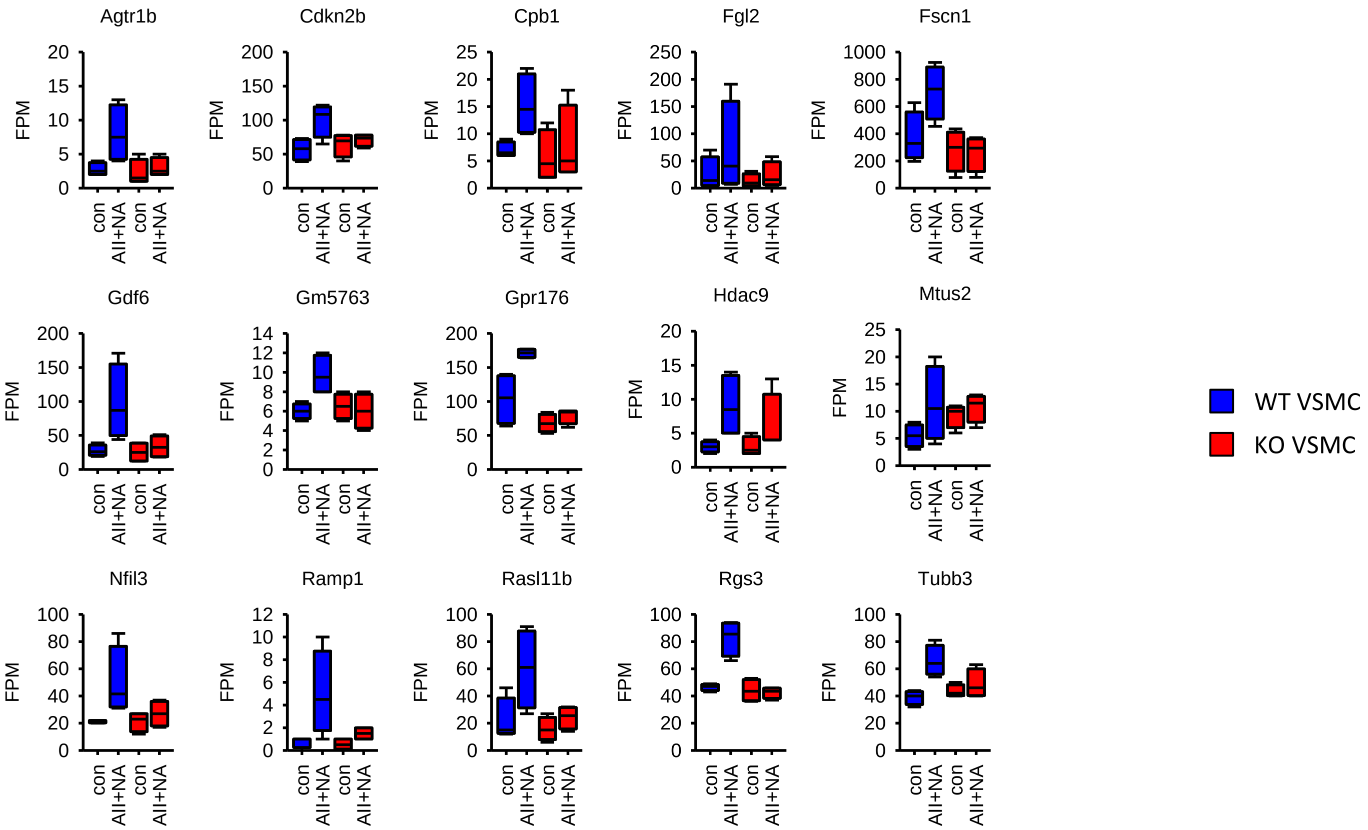

SF5C. FPM values for the 15 DEG affected by humoral stressors (angiotensin II + noradrenaline, AII+NA) in WT VSMC. These genes were not affected in KO VSMC. For each cell type and each condition 4 independent biological replicates were included in the differential expression analysis.

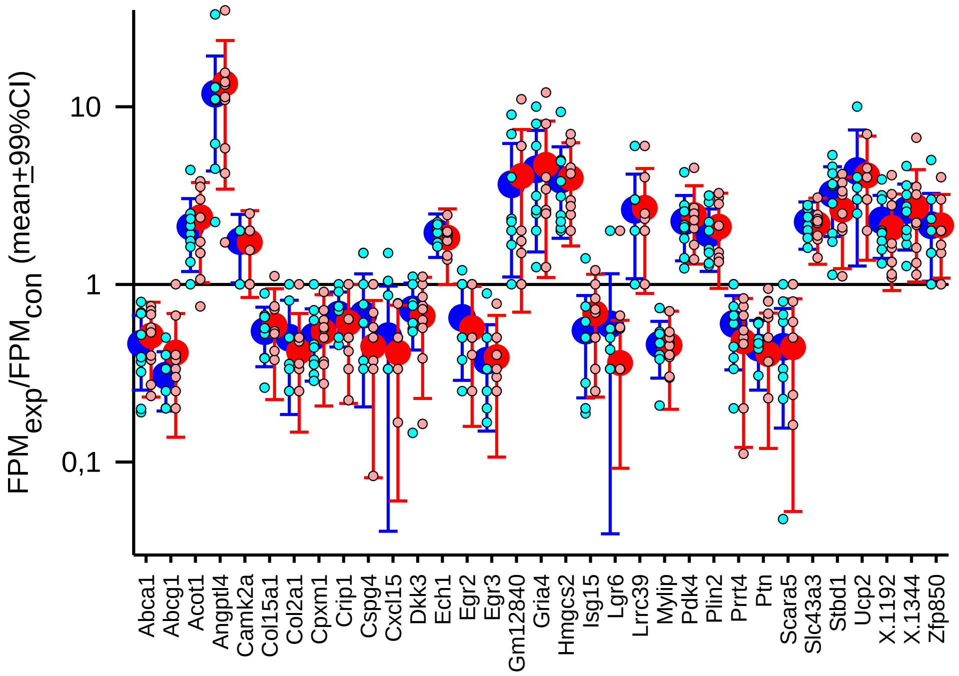

EC (|log2FC| > 1.0; FDR < 0.05)	
**[DEG]**	**Overlap**
**HG+FFS**	15 ↑, 13 ↓
**All+NA**	0
**ALL**	18 ↑, 12 ↓

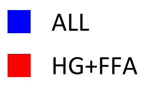

SF6. DEG affected by exposure of endothelial cells (EC) to metabolic stressors or all stressors with a log_2FC threshold of 0.9 and an overlap of FDR thresholds = 0.05 for DESeq2 and edgeR. For each condition 7 independent biological replicates were included in the differential expression analysis.

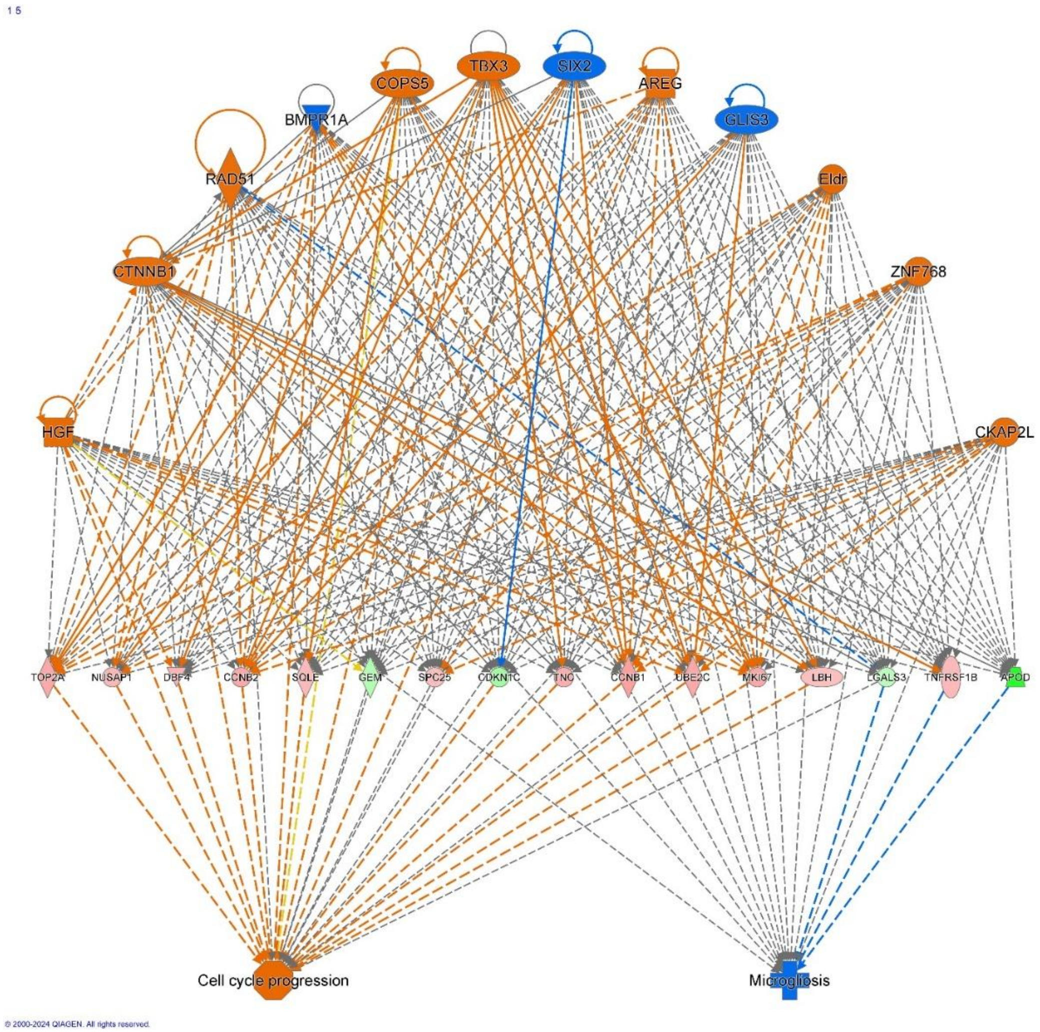

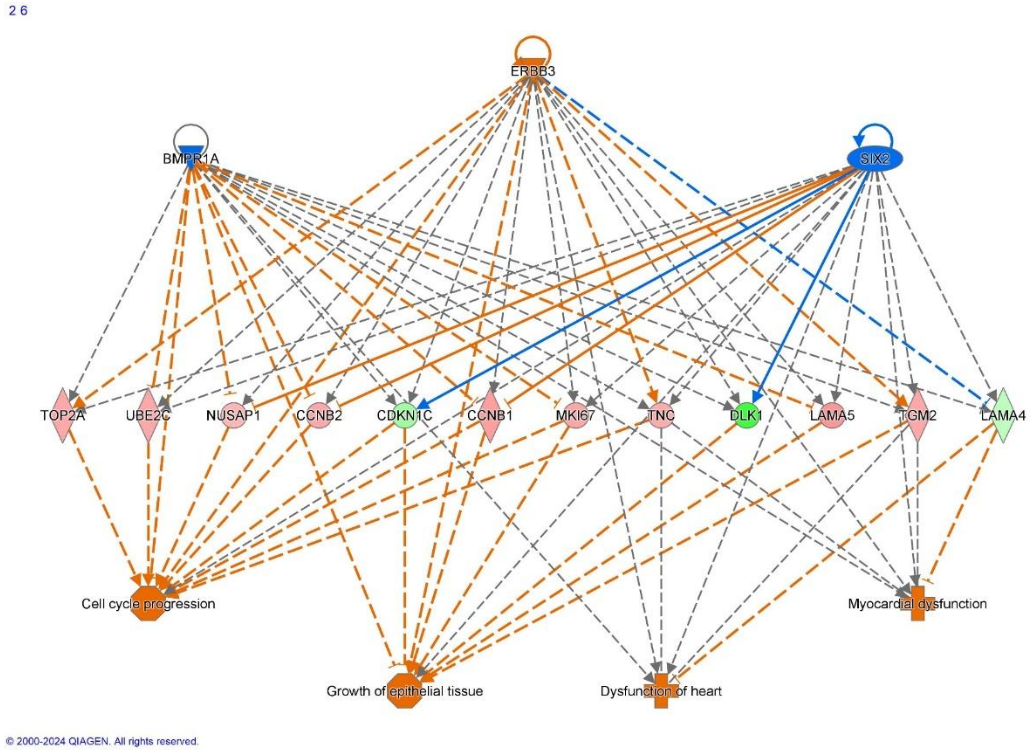

SF7. Two regulator effects networks (RE) predicted by IPA with high consistency scores, based on the results obtained for WT VSMC exposed to all stressors. These networks link the considered DEGwith predicted upstream regulators and associated downstream events

cluster		gene count	primary description
1	🔴	32	Mixed, incl. Cellular response to interferon-beta, and Antiviral mechanism by IFN-stimulated genes
2	🟠	28	Mixed, incl. Positive regulation of chromosome separation, and Kinesin motor, catalytic domain. ATPase.
3	🟤	13	ECM-receptor interaction
4	🟡	9	Regulation of systemic arterial blood pressure by hormone
5	🟢	8	RA biosynthesis pathway
6	🟢	7	Sterol biosynthesis
7	🟢	7	Assembly of collagen fibrils and other multimeric structures

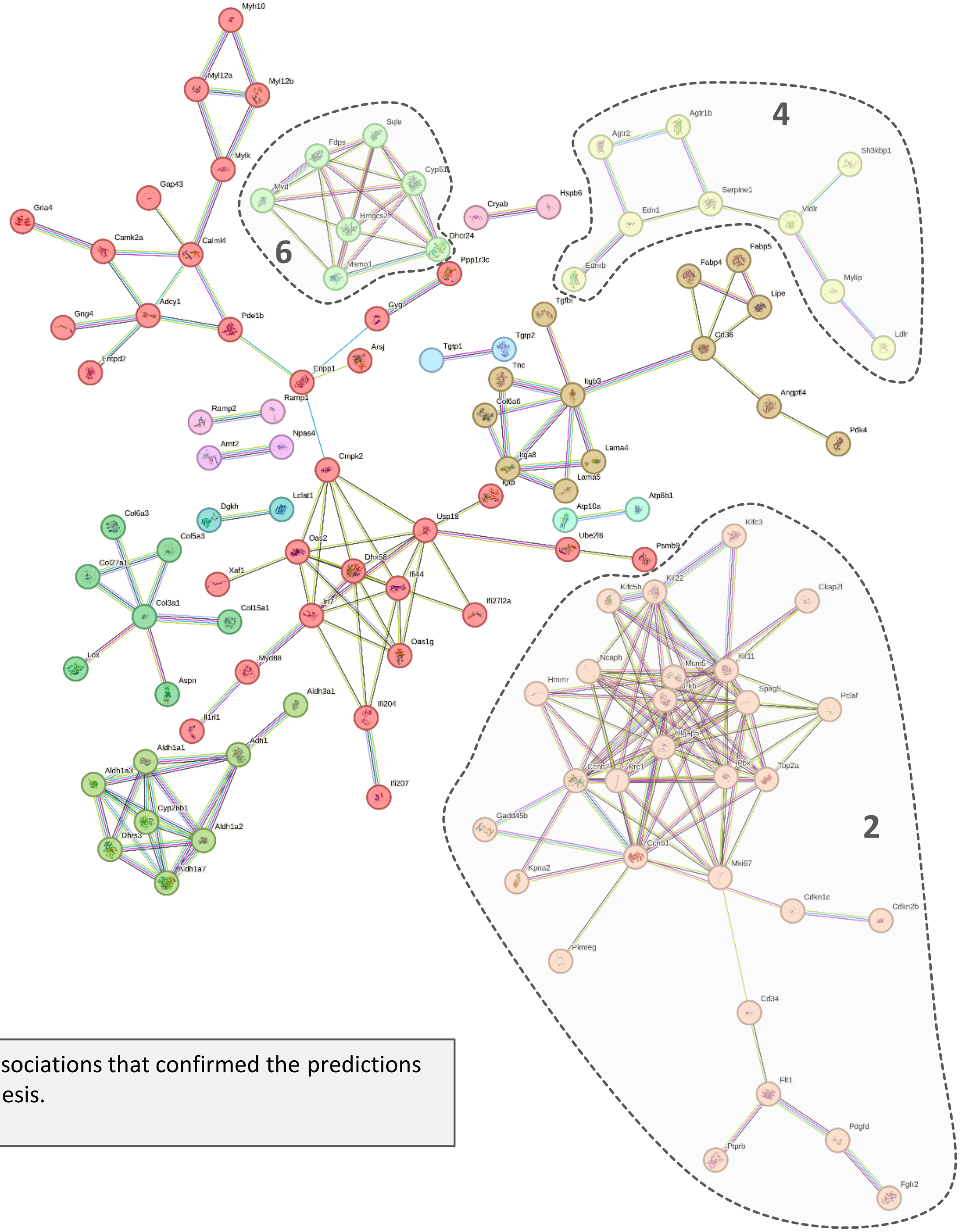

SF8. Protein network analysis by STRING (using DEG induced by all stressors in WT VSMC) predicted associations that confirmed the predictions of the IPA analysis, identifying clusters a.o. for cell proliferation, vessel contraction and sterol biosynthesis.

Ingenuity Canonical Pathways	$-log_{10}$(p-value)	z-score
N/A		

Diseases and Bio Functions, Categories	Functions Annotation	$-log_{10}$(p-value)	z-score
Free Radical Scavenging	Production of reactive oxygen species	1,91	-2,56
Free Radical Scavenging	Synthesis of reactive oxygen species	2,32	-2,22
Lipid Metabolism	Quantity of steroid	4,05	-2,22

Upstream Regulator	Molecule Type	$-log_{10}$(p-value)	z-score
PPARA	ligand-dependent nuclear receptor	7,75	3,04
KMT2D	transcription regulator	3,99	2,63
palmitic acid	chemical - endogenous mammalian	3,13	2,22
linoleic acid	chemical - endogenous mammalian	4,12	2,19
YAP1	transcription regulator	4,56	2,06
oleic acid	chemical - endogenous mammalian	5,90	2,02
D-glucose	chemical - endogenous mammalian	3,98	-2,06
STING1	ion channel	2,75	-2,13
IFNB1	cytokine	2,77	-2,17
PRL	cytokine	2,17	-2,22
IL5	cytokine	2,36	-2,22
PML	transcription regulator	7,38	-2,81

SF9. Results of IPA analysis using DEG resulting from the exposure of EC to all stressors. IPA predicts in principle only the expected cellular reaction to metabolic stressors and no effect of humoral stressors. IPA analysis using DEG resulting from the exposure of EC to metabolic stressors gave similar results.

Early-phase impact of obesity-associated stress on murine vascular smooth muscle cells depends on EGFR and sex

z-scores for Canonical Pathways (B-H p-value < 0.05)	WT VSMC, ALL	WT VSMC, HG+FFA	KO VSMC, ALL	KO VSMC, HG+FFA	EC, ALL	EC, HG+FFA
Regulation of lipid metabolism by PPARalpha	1,34		2,24		1,34	1,34
Pathogen Induced Cytokine Storm Signaling Pathway	-2,31				-2,00	
Wound Healing Signaling Pathway	-2,11					
Collagen biosynthesis and modifying enzymes	-2,65					
Collagen chain trimerization	-2,65					
Collagen degradation	-2,45					
Role of Osteoclasts in Arthritis Signaling Pathway	-2,53					
GP6 Signaling Pathway	-2,53					
CREB Signaling in Neurons	2,14			2,00		
Superpathway of Cholesterol Biosynthesis	2,65					
Cholesterol biosynthesis	2,45					
Cohesin Chromatin Regulation Pathway	2,24					
Cardiac Hypertrophy Signaling (Enhanced)	2,11					
Smooth Muscle Contraction	2,00					
Cholesterol Biosynthesis III (via Desmosterol)	2,00					
Activation of gene expression by SREBF (SREBP)	2,00					
Synaptic Long Term Potentiation	2,00					
Cholesterol Biosynthesis II (via 24,25-dihydrolanosterol)	2,00					
Activation of NMDA receptors and postsynaptic events	2,00					
Cholesterol Biosynthesis I	2,00					

z-scores for Diseases & Biofunctions (B-H p-value < 0.05)	WT VSMC, ALL	WT VSMC, HG+FFA	KO VSMC, ALL	KO VSMC, HG+FFA	EC, ALL	EC, HG+FFA
Apoptosis	-2,02	-0,23	-0,45	0,63	-1,95	-0,56
Fatty acid metabolism	1,92		1,02	2,24	-0,58	-0,70
Proliferation of neural cells	2,01			1,31	0,04	0,16
Proliferation of connective tissue cells	2,05	1,54				
Cell movement	3,27	1,65			-0,09	-0,34
Migration of cells	3,48	1,27			-0,41	-0,47
Vasculogenesis	2,82	0,59	-0,28	-0,37		-1,25
Atherosclerosis	2,15		-0,93		0,35	0,67
Growth of vessel	2,53		-1,18			
Hydrolysis of lipid	2,49				1,26	
Cell viability	2,18	-0,19	-0,45	-0,30	0,98	
Metabolism of carbohydrate	2,50		0,98		0,58	
Growth of neurites	2,50		0,98		-0,29	
Proliferation of neuronal cells	2,45				-0,05	-0,49
Migration of endothelial cells	2,28					-0,74
Proliferation of muscle cells	2,78		-0,25			
Vascularization	2,91					
Protein kinase cascade	2,81					
Growth of muscle tissue	2,78					
Synthesis of carbohydrate	2,69					
Outgrowth of neurites	2,72					
MAPKKK cascade	2,73					
Outgrowth of vessel	2,43					
Formation of actin stress fibers	2,38					
Synthesis of inositol phosphate	2,37					
Neovascularization	2,40					
Proliferation of smooth muscle cells	2,39					
Formation of cytoskeleton	2,62					
Outgrowth of cells	2,49					
Formation of filaments	2,53					
Fibrogenesis	2,55					
Synthesis of polyols	2,56					
Quantity of carbohydrate	1,23	0,43	-0,17	2,16	1,08	1,08
Necrosis	-1,09	0,15	-2,04		-1,04	0,66
Oxidation of lipid	1,63	0,96	2,01			
Organization of cytoskeleton	2,02	0,00	2,45			
Production of reactive oxygen species			-0,90		-2,56	
Synthesis of reactive oxygen species					-2,22	
Angiogenesis	2,63				-1,39	-0,86
Quantity of steroid	2,18		0,30	0,15	-2,22	-0,67
Failure of heart	-2,43					
Expansion of cells			0,93			-2,19
Glucose tolerance	0,01		-0,31			-2,45
Vaso-occlusion	2,15				0,35	
Metabolism of polysaccharide	2,28					
Cell movement of muscle cells	2,11					
Occlusion of artery	2,15					
Occlusion of blood vessel	2,15					
Invasion of cells	2,17					
Contraction of cells	2,03					
Growth of connective tissue	2,05					
Mineralization of bone	2,05					

z-scores for Upstream Regulators (B-H p-value < 0.05)	WT VSMC, ALL	WT VSMC, HG+FFA	KO VSMC, ALL	KO VSMC, HG+FFA	EC, ALL	EC, HG+FFA
PPARA	1,25	2,18	2,90	2,36	3,04	2,88
ERBB2	2,58	1,28	0,88	-1,18	0,16	0,30
VEGF	3,47	0,82	1,00		-0,44	-1,01
hydrogen peroxide	2,92	1,07	1,03		-0,04	-0,26
beta-estradiol	3,35	1,35	-0,40	0,05	0,53	-0,73
TBX3	3,59	2,22				
Eldr	3,00	2,00				
KMT2D	1,32				2,63	0,25
NFAT5	2,54				1,11	
F3	3,09				1,26	
ACKR2	2,83				2,00	
CEBPB	2,53	1,10	1,70		1,91	2,19
l-asparaginase	-3,50					
POR	-2,76				1,00	1,00
PML	-1,72				-2,81	-2,24
TCF7	-2,52					-2,00
IRF3	-3,52				-1,99	
IRF7	-3,54				-1,96	
STING1	-2,93				-2,13	
MAVS	-2,63				-1,95	
SRF	2,68				-1,93	-1,94
SREBF1	2,54		-1,37		0,31	-0,36
Ca2+	2,55				-1,11	
CREB1	3,10		0,49		0,56	
QKI	3,27					
MYOD1	2,98					
HMG20A	2,89					
TREX1	3,07					
SREBF2	3,10					
PNPT1	3,13					
RXRA	2,70				-0,39	
PTGER4	2,72					
SCAP	2,75					
F2	2,76					
RNASEH2B	2,55					
TBK1	2,54					
KLF5	2,59					
TRIM24	2,69					
TCR (complex)	2,62					
AREG	2,63					
SMARCAL1	2,65					
ETNPPL	2,65					
RABL6	2,65					
ATP7B	2,65					
IFNAR	-2,55					
KDM5B	-2,67					
APP	-2,63					
ZBTB10	-2,76					

SF10. IPA comparative analysis for the three cell types, exposed either to all stressors or to metabolic stressors for canonical pathways, disease and biofunctions and upstream regulators.

Early-phase impact of obesity-associated stress on murine vascular smooth muscle cells depends on EGFR and sex

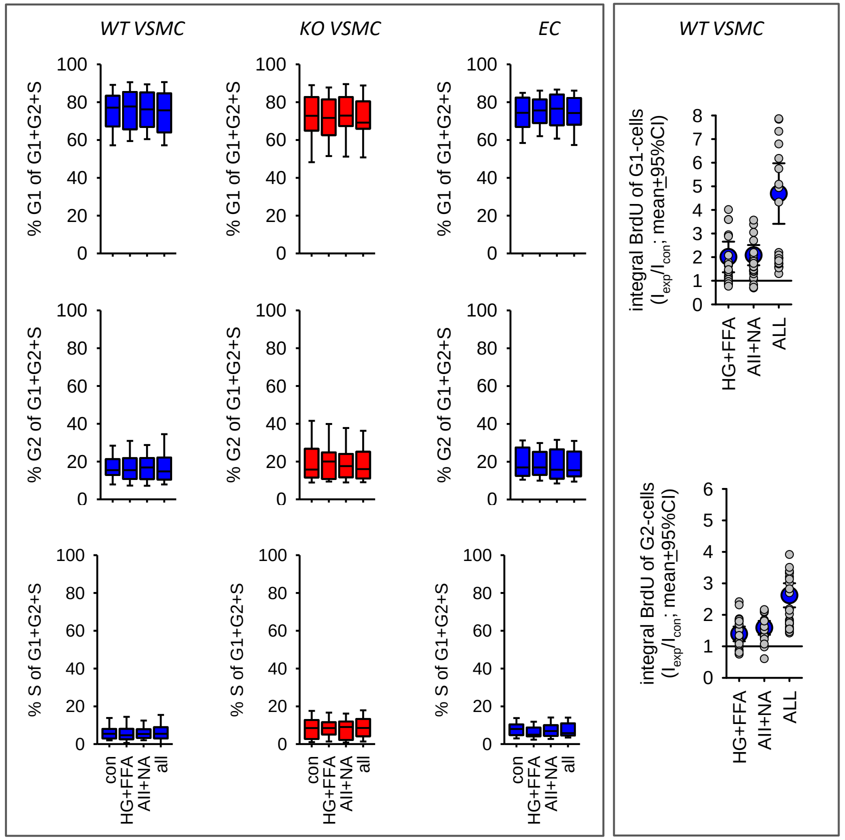

SF11. Relative distribution of cells to G1-, S- and G2-phase of the cell cycle under control conditions and after exposure to the different stressor conditions. The right panel shows the impact of the stressors on nuclear BrdU-incorporation in WT VSMC from G1- and G2-phase. N = 7 plates with 5 wells for each condition per cell type.

Early-phase impact of obesity-associated stress on murine vascular smooth muscle cells depends on EGFR and sex

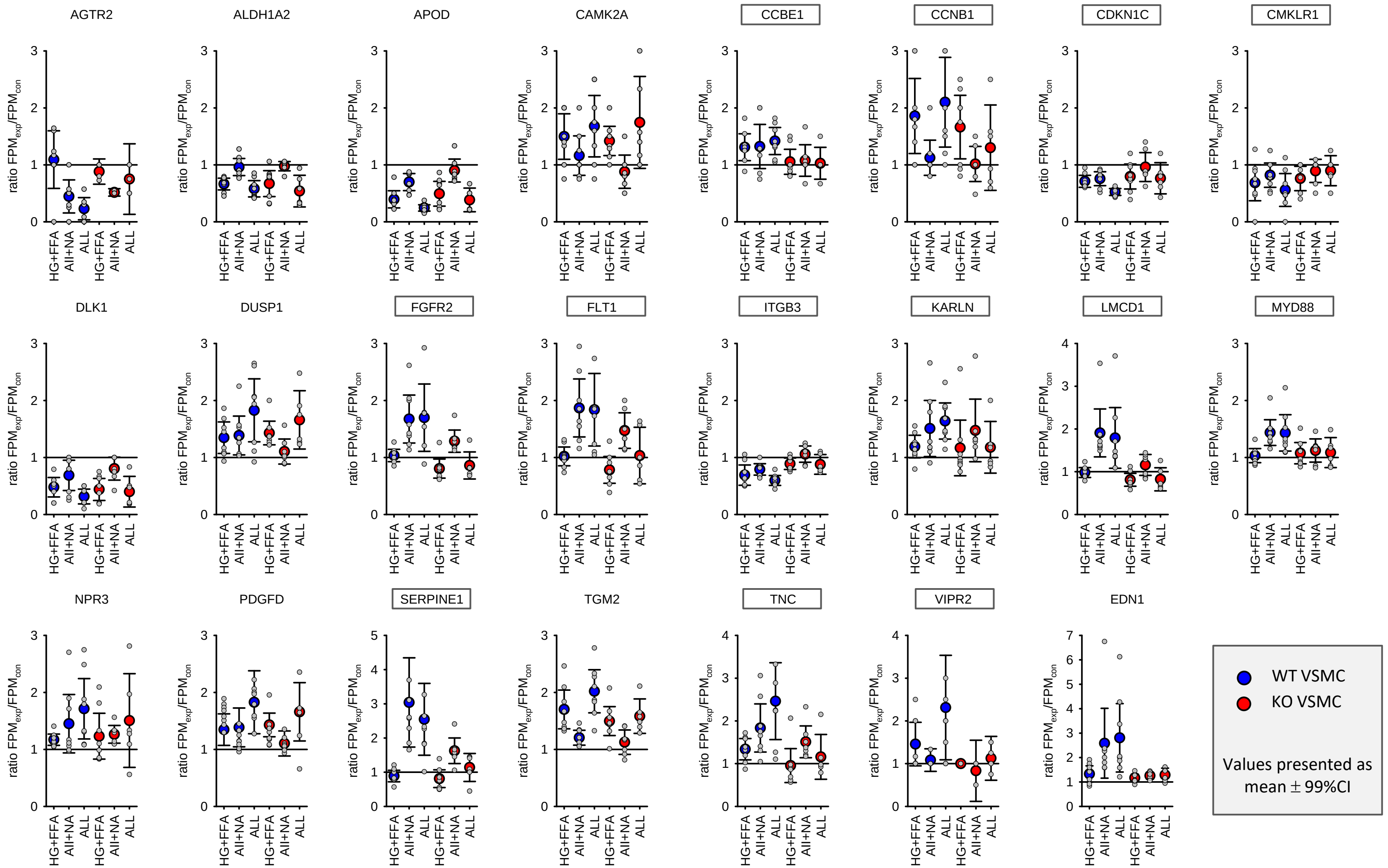

DEG in DBF: proliferation of muscle cells (expression log ratio: log_2FC)							
**Symbol**	**Entrez Gene Name**	**EC, ALL**	**EC, HG+FFA**	**WT VSMC, ALL**	**WT VSMC, HG+FFA**	**KO VSMC, ALL**	**KO VSMC, HG+FFA**
AGTR2	angiotensin II receptor type 2			-1,45			
ALDH1A2	aldehyde dehydrogenase 1 family member A2		-0,77	-0,80			
APOD	apolipoprotein D			-1,96	-1,67		
CAMK2A	calcium/calmodulin dependent protein kinase II alpha			0,75			
CCBE1	collagen and calcium binding EGF domains 1			0,68			
CCNB1	cyclin B1			0,92	0,91		
CDKN1C	cyclin dependent kinase inhibitor 1C			-0,85			
CMKLR1	chemerin chemokine-like receptor 1			-1,10			
DLK1	delta like non-canonical Notch ligand 1			-1,39		-1,68	
DUSP1	dual specificity phosphatase 1			0,92			
EDN1	endothelin 1			1,67			
FGFR2	fibroblast growth factor receptor 2			0,80			
FLT1	fms related receptor tyrosine kinase 1			0,81			
ITGB3	integrin subunit beta 3			-0,93			
KALRN	kalirin RhoGEF kinase			0,68			
LMCD1	LIM and cysteine rich domains 1			1,00			
MYD88	MYD88 innate immune signal transduction adaptor			0,72			
MYH10	myosin heavy chain 10			1,16			
NPR3	natriuretic peptide receptor 3	0,65	0,67	0,74			
PDGFD	platelet derived growth factor D			0,92	0,67		
SERPINE1	serpin family E member 1			1,45			
TGM2	transglutaminase 2	1,21	1,08	1,06	0,85	0,79	0,74
TNC	tenascin C			1,49			
VIPR2	vasoactive intestinal peptide receptor 2			1,01			

SF12. Impact of the stressors on the genes contained in the IPA terms for proliferation for WT VSMC and KO VSMC. For each cell type and each condition up to 8 independent biological replicates were included in the analysis.

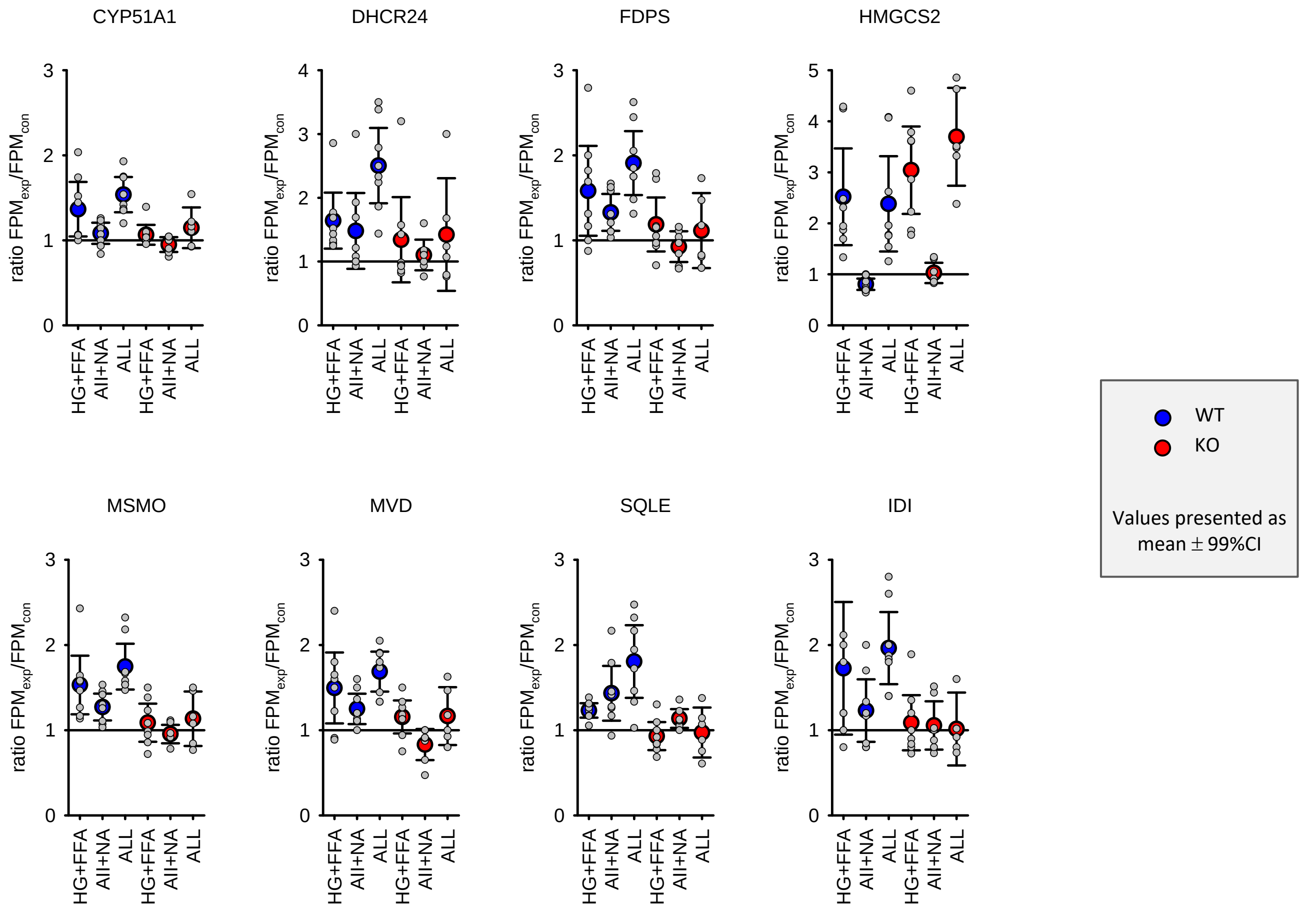

SF13. Impact of the stressors on the genes contained in the IPA terms for lipid metabolism for WT VSMC and KO VSMC. For each cell type and each condition up to 8 independent biological replicates were included in the analysis.

Early-phase impact of obesity-associated stress on murine vascular smooth muscle cells depends on EGFR and sex

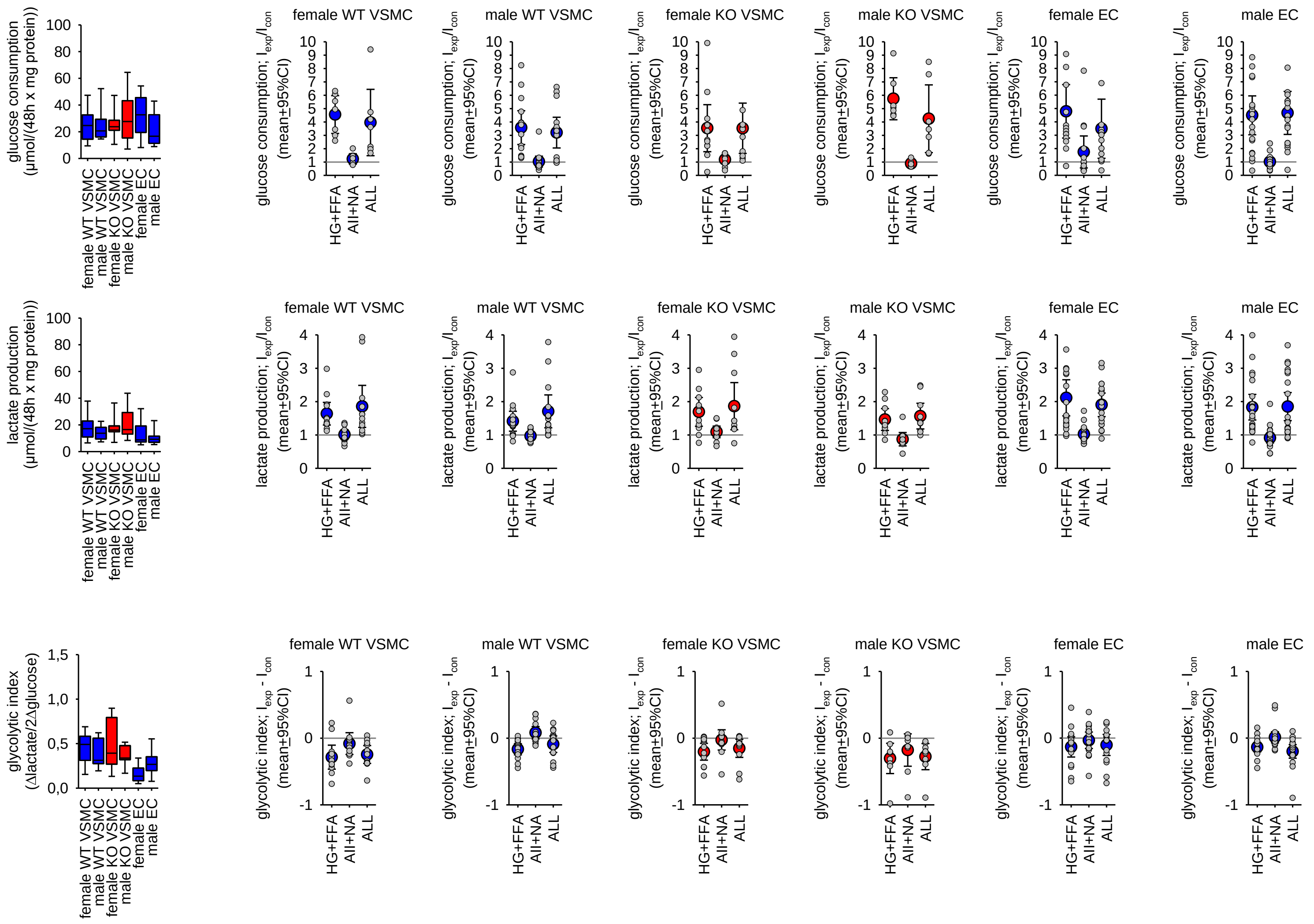

SF14. Impact of the stressors on glucose consumption, lactate production and the glycolytic index (Δlactate/2×Δglucose) of male and female VSMC as well as EC. N ≥ 7 independent sets of samples.

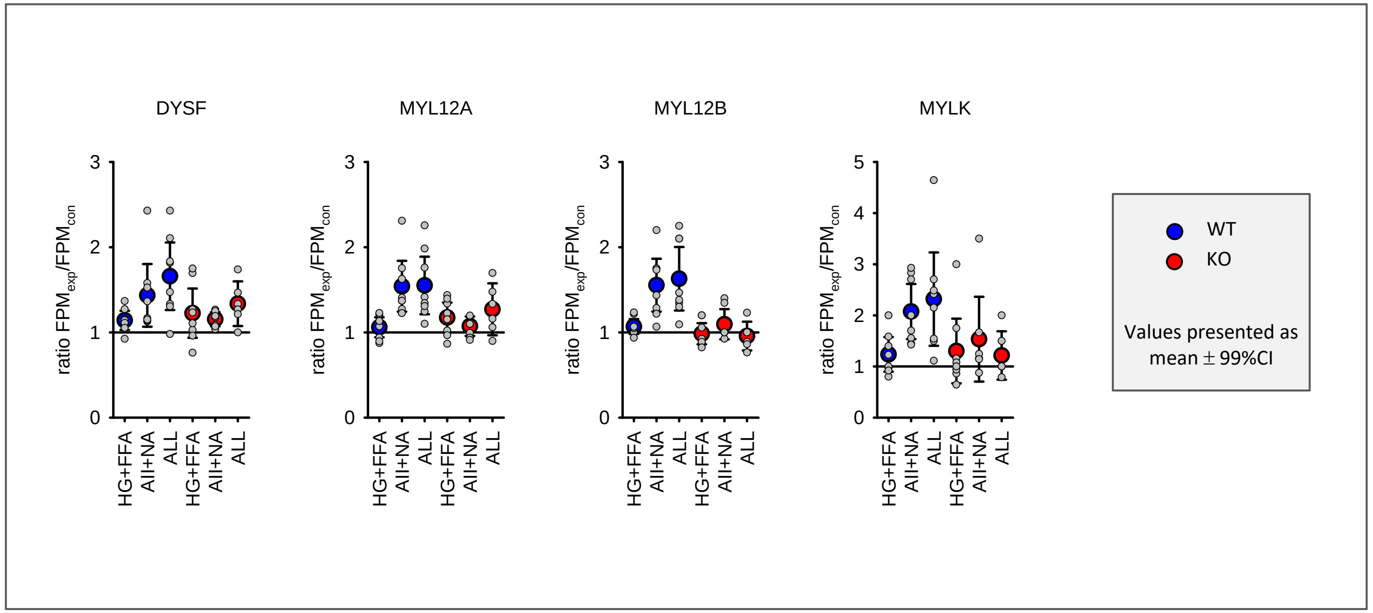

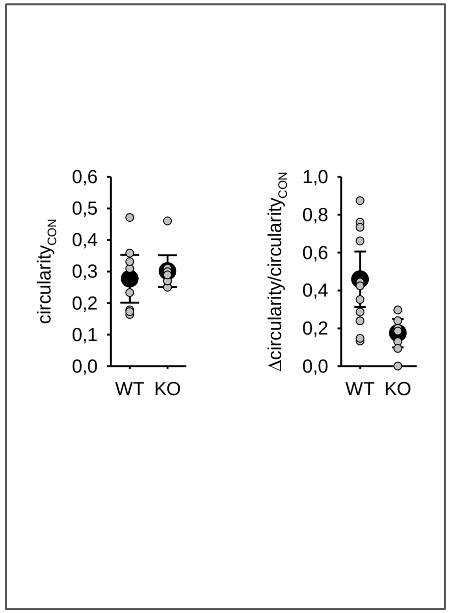

SF15. Impact of the stressors on the genes contained in the IPA terms related to cell contraction for WT VSMC and KO VSMC. For each cell type and each condition up to 8 independent biological replicates were included in the analysis. The right panel shows the comparison of control circularity and Ca^{2+}-induced changes in circularity for WT VSMC and KO VSMC. N = 7-10 plates with 6 wells for each condition.

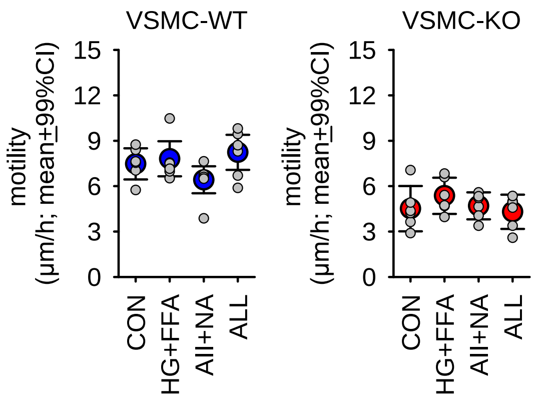

SF16. Spontaneous random motility of WT VSMC and VSMC-KO cells. N = 6 wells for each condition per cell type.

Early-phase impact of obesity-associated stress on murine vascular smooth muscle cells depends on EGFR and sex

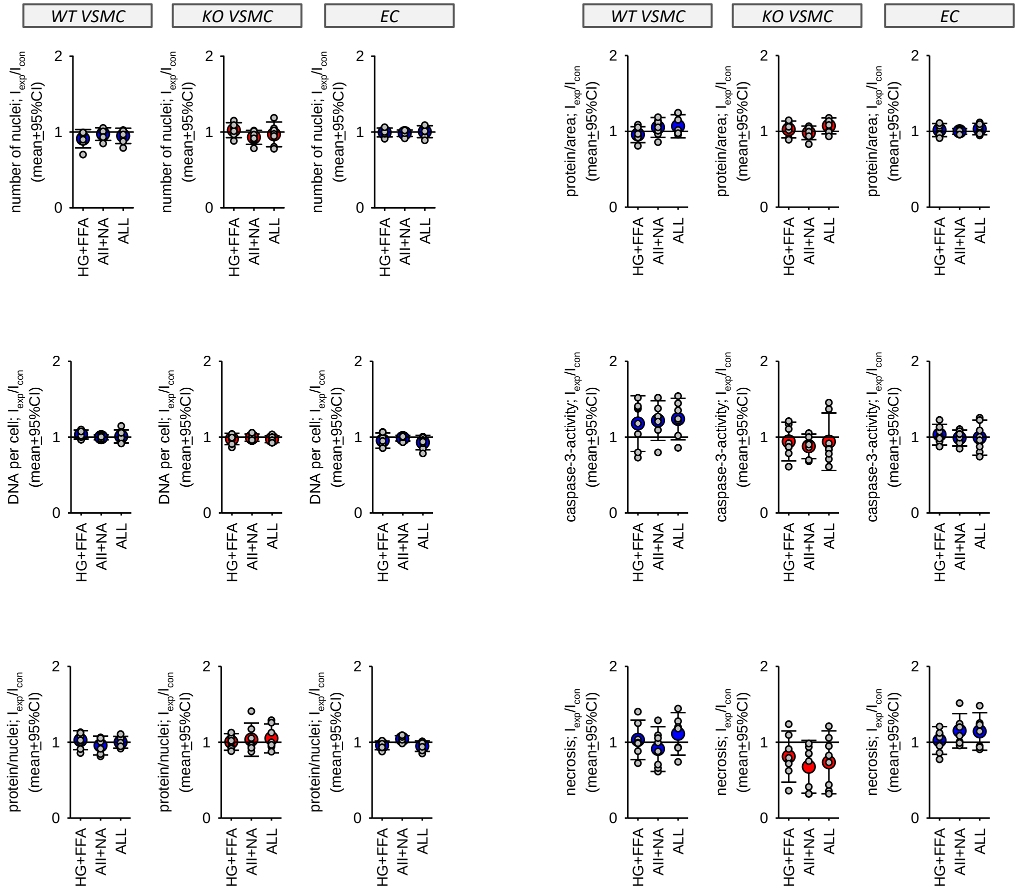

SF17. Effect of the stressors on the number of nuclei (i.e. number of cells), nuclear DNA content, cellular protein, the apoptosis marker caspase-3-activity and necrosis (determined by trypan blue exclusion) of female cells. N = 8 plates with up to 6 wells for each condition and each cell type.

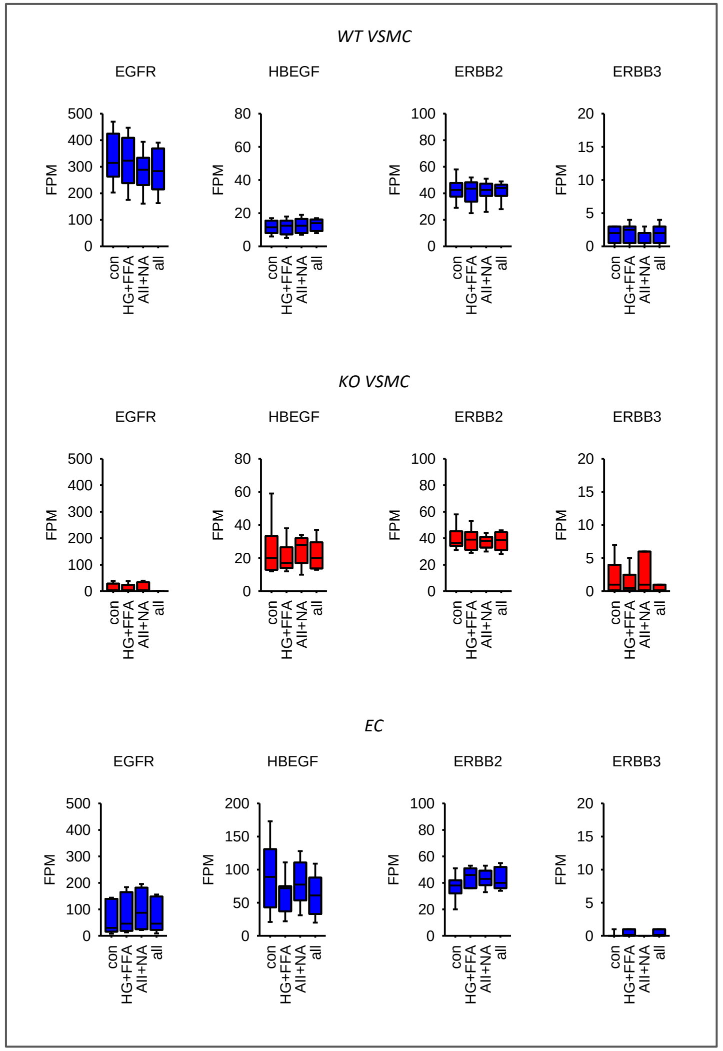

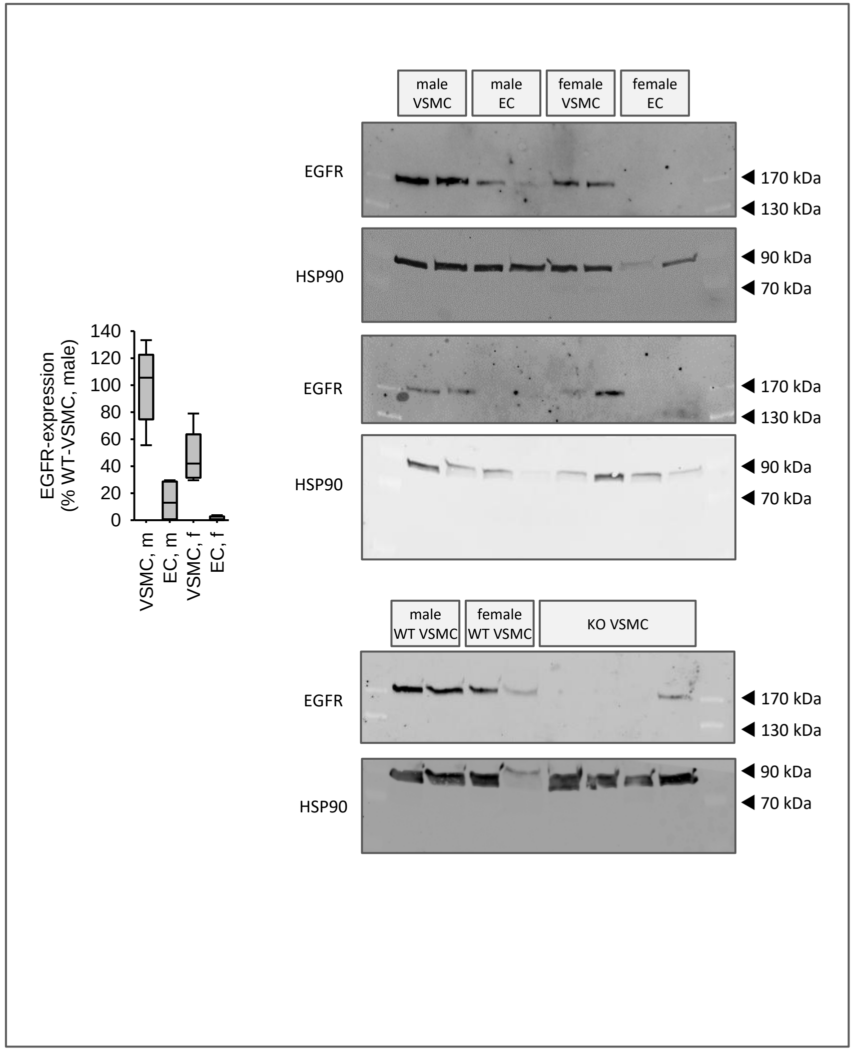

Immunoblotting

For protein expression level determination, cells were lysed with CST lysis buffer (20mM Tris, pH 7.5 (Illinois Tools Works companies), 150mM NaCl (Roth), 1% Triton X-100 (Sigma-Aldrich), 1mM EDTA (Merck), 1mM EGTA (Sigma-Aldrich), 184mg/L Na-orthovanadate (Sigma-Aldrich), 2.5mM Na-pyrophosphate (Sigma-Aldrich), 1mM β-glycerolphosphate (Sigma-Aldrich)), centrifuged at 13.000 g for 10 minutes and protein amount was determined with Bradford assay.

Equal amounts of the proteins were denaturated with 6x Laemmli buffer (0.5M Tris pH 6.8 (Roth GmbH), 10% SDS (Roth), 10% Glycerol (Sigma-Aldrich)) at 95°C for 5-10 minutes. Proteins were separated by 10% sodium dodecyl sulfate-polyacrylamide gel electrophoresis (SDS-PAGE) and transferred onto a nitrocellulose membrane.

After blocking with 5 % nonfat dry milk powder in Tris-buffered saline with Tween20 (TBS-Tween) (20mM Tris base, pH 7.4 (Illinois Tools Works companies), 150mM NaCl, 0.05% Tween-20 (Sigma-Aldrich)) membranes were incubated with first antibody (Cell Signaling Technologies, Frankfurt, Germany; EGFR XP #4267, 1:2000) diluted in 5 % bovine serum albumin (BSA) in TBS-Tween overnight. Horse radish peroxidase (HRP)-coupled secondary antibodies, Anti-Rabbit IgG HRP, Cell Signaling #7074, 1:1000 in 5 % nonfat dry milk powder in TBS-Tween) were used. After removal of unbound secondary antibody three washing steps in TBS-TWEEN were performed.

Finally Clarity™ Western ECL Substrate (Bio-Rad, Munich, Germany) was added and the peroxidase activity-based light emission was recorded by an imaging system (Image Quant LAS4000, GE Health care, Buckinghamshire, GB). Densitometry analysis was performed with Quantity One software from BioRad (Feldkirchen, Germany) and the relative expression values calculated under consideration of membrane protein staining signal (Ponceau).

SF18. Left panel: Impact of the stressors on the RNA abundance of members of the EGFR family (EGFR, ERBB2, ERBB3) and the membrane-bound ligand HBEGF in male WT VSMC, KO VSMC and EC. For each cell type and each condition 4 independent biological replicates were included in the analysis. Right panel: EGFR protein abundance in male and female WT VSMC and EC.

Early-phase impact of obesity-associated stress on murine vascular smooth muscle cells depends on EGFR and sex

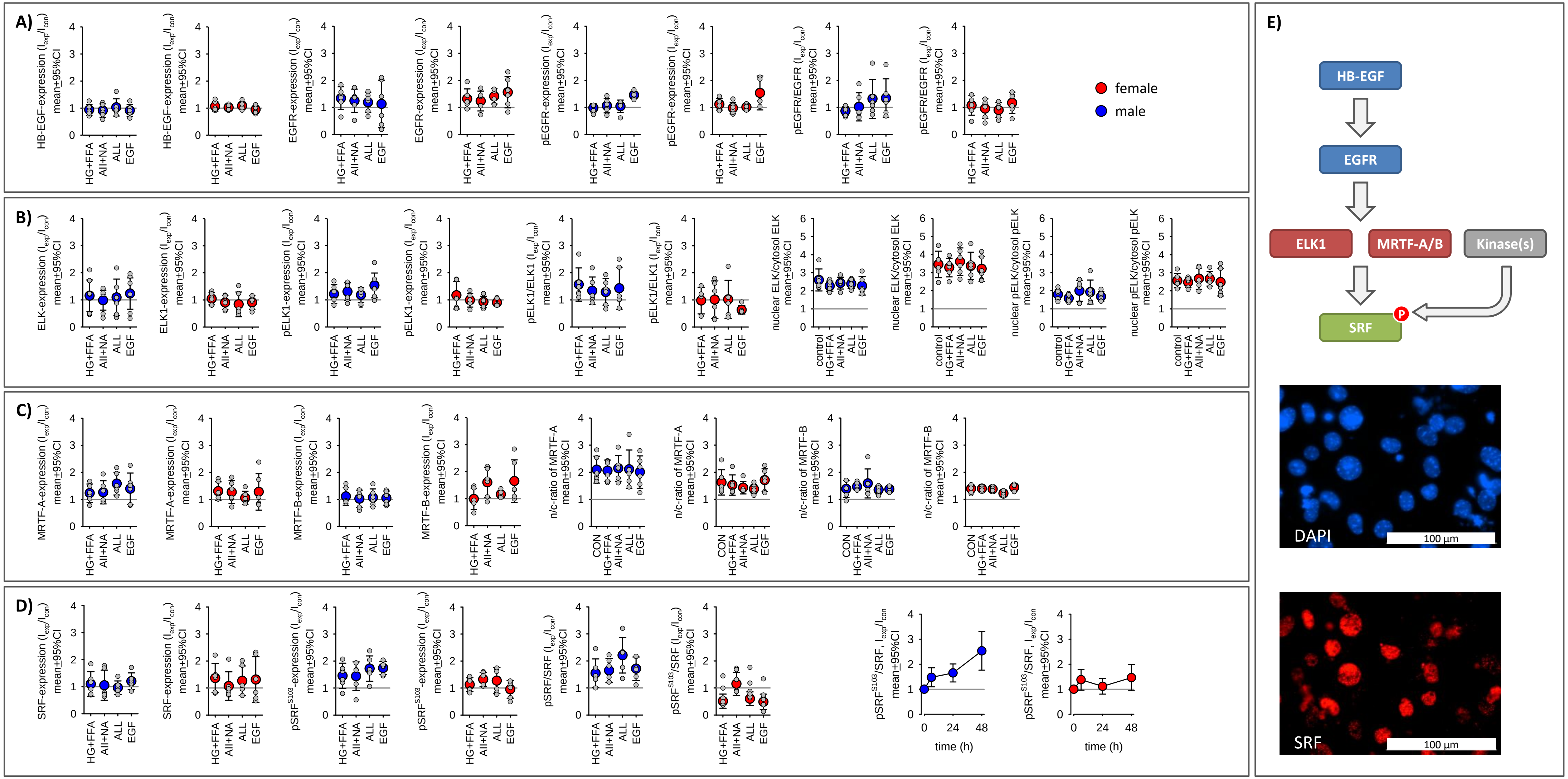

SF19. Expression of different components of the EGFR-SRF pathway in male and female WT VSMC. A) Ex-pression of HB-EGF, EGFR and phospho-EGFR^{Y1086} after 48 h exposure to the stressors. B) Expression and nucleus-to-cytosol distribution of ELK1 and phosphor-ELK1^{S383} after 48 h exposure to the stressors. C) Expression and nucleus-to-cytosol distribution of MRTF-A or MRTF-B after 48 h exposure to the stressors. D) Expression of SRF and phsopho-SRF^{S103} after 48 h exposure to the stressors. The two panels on the right show the time course of enhanced phsopho-SRF^{S103} in the presence of ALL stressors in male WT VSMC. N = 6 plates with 3 wells each. E) Scheme of SRF-activation and immunofluorescent image of nuclear SRF.

Supplementary figure SF20. Original blot images.

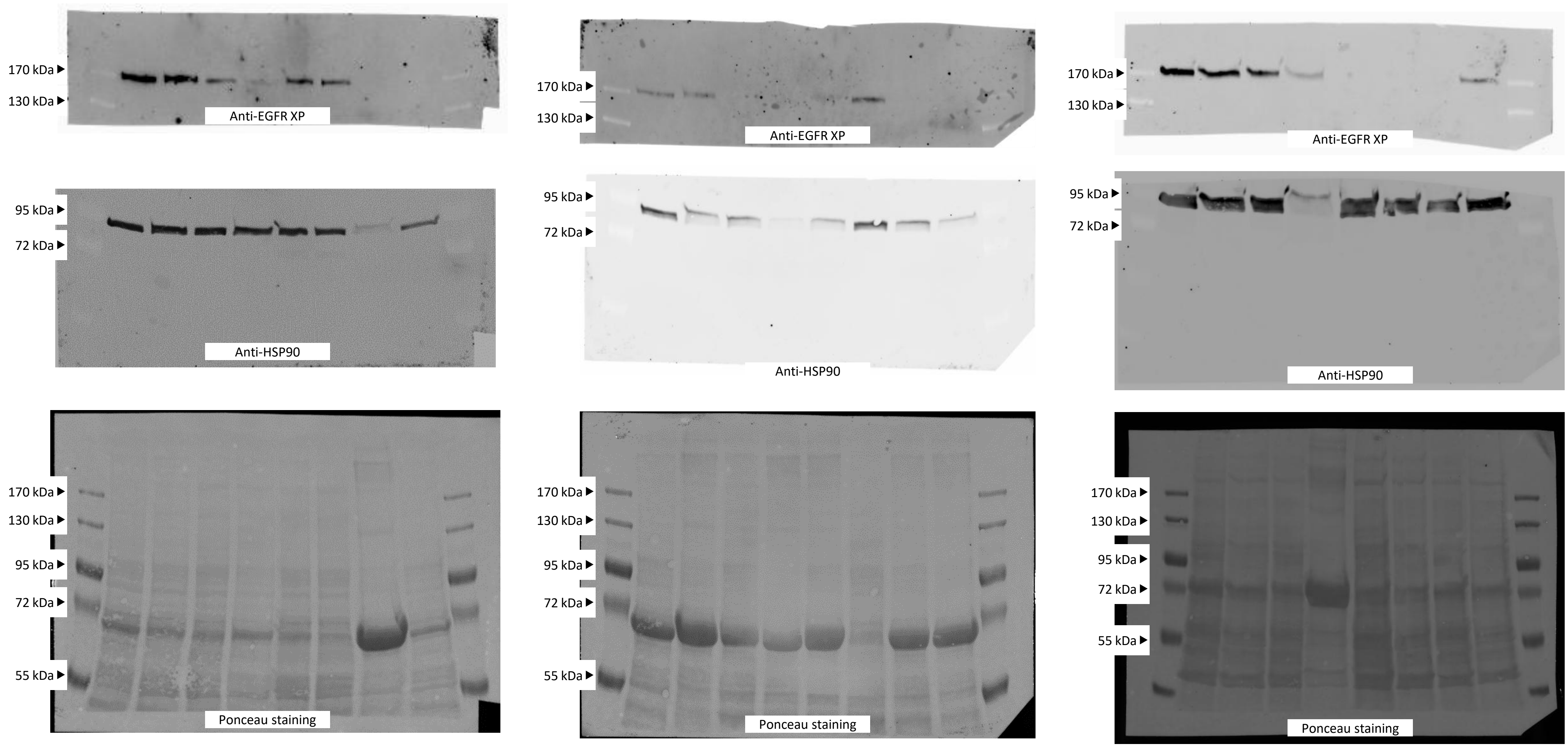

© Michael Gekle | Julius-Bernstein-Institut für Physiologie | Martin-Luther-Universität Halle-Wittenberg

Supplementary data

Supplementary Data 1
Effect of stressors on canonical biomarker expression in VSMC-WT, VSMC-KO or EC.
Supplementary Data 2
IPA analysis results for canonical pathways (CP) of cells exposed to all stressors. Comparison_VSMC-WT_VSMC-KO_EC.
Supplementary Data 3
IPA analysis results for canonical pathways (CP) of cells exposed to all stressors. VSMC-WT.
Supplementary Data 4
IPA analysis results for canonical pathways (CP) of cells exposed to all stressors. VSMC-KO.
Supplementary Data 5
IPA analysis results for canonical pathways (CP) of cells exposed to all stressors. EC.
Supplementary Data 6
IPA analysis results for disease and biofunctions (DBF) of cells exposed to all stressors. Comparison_VSMC-WT_VSMC-KO_EC.
Supplementary Data 7
IPA analysis results for disease and biofunctions (DBF) of cells exposed to all stressors. VSMC-WT.
Supplementary Data 8
IPA analysis results for disease and biofunctions (DBF) of cells exposed to all stressors. VSMC-KO.
Supplementary Data 9
IPA analysis results for disease and biofunctions (DBF) of cells exposed to all stressors. EC.
Supplementary Data 10
IPA analysis results for upstream regulator analysis (URA) of cells exposed to all stressors. Comparison_VSMC-WT_VSMC-KO_EC.
Supplementary Data 11
IPA analysis results for upstream regulator analysis (URA) of cells exposed to all stressors. VSMC-WT.
Supplementary Data 12
IPA analysis results for upstream regulator analysis (URA) of cells exposed to all stressors. VSMC-KO.
Supplementary Data 13
IPA analysis results for upstream regulator analysis (URA) of cells exposed to all stressors. EC.
Supplementary Data 14
Results of gene ontology enrichment analysis with g:Profiler. VSMC-WT.
Supplementary Data 15
Results of gene ontology enrichment analysis with g:Profiler. VSMC-KO.
Supplementary Data 16
Masterfiles with all FPM values. VSMC.
Supplementary Data 17
Masterfiles with all FPM values. EC.
Supplementary Data 18
Lists of genes for enrichment analysis. VSMC-WT, ALL versus CON.
Supplementary Data 19
Lists of genes for enrichment analysis. VSMC-WT, HG+FFA versus CON.
Supplementary Data 20
Lists of genes for enrichment analysis. VSMC-KO, ALL versus CON.
Supplementary Data 21
Lists of genes for enrichment analysis. VSMC-KO, HG+FFA versus CON.
Supplementary Data 22
Lists of genes for enrichment analysis. EC, ALL versus CON.
Supplementary Data 23
Lists of genes for enrichment analysis. EC, HG+FFA versus CON.
Supplementary Data 24
Numerical source data of the figures, unless these data are already part of supplementary data.
