# Supplementary material for: Early-phase impact of obesity-associated stress on murine vascular smooth muscle cells depends on EGFR and sex
Source: Commun Biol. 2025 Dec 22;8:1834. doi: 10.1038/s42003-025-09416-7 (PMC12749158; doi:10.1038/s42003-025-09416-7)
Supplement: Supplementary file 2 — Description of Additional Supplementary Files [file 42003_2025_9416_MOESM2_ESM.docx]

Description of Additional Supplementary File

File name: Supplementary Data 1
Description: Effect of stressors on canonical biomarker expression in VSMC-WT, VSMC-KO or EC.

File name: Supplementary Data 2
Description: IPA analysis results for canonical pathways (CP) of cells exposed to all stressors. Comparison_VSMC-WT_VSMC-KO_EC.

File name: Supplementary Data 3
Description: IPA analysis results for canonical pathways (CP) of cells exposed to all stressors. VSMCWT.

File name: Supplementary Data 4
Description: IPA analysis results for canonical pathways (CP) of cells exposed to all stressors. VSMCKO.

File name: Supplementary Data 5
Description: IPA analysis results for canonical pathways (CP) of cells exposed to all stressors. EC.

File name: Supplementary Data 6
Description: IPA analysis results for disease and biofunctions (DBF) of cells exposed to all stressors. Comparison_VSMC-WT_VSMCKO_EC.

File name: Supplementary Data 7
Description: IPA analysis results for disease and biofunctions (DBF) of cells exposed to all stressors. VSMC-WT.

File name: Supplementary Data 8
Description: IPA analysis results for disease and biofunctions (DBF) of cells exposed to all stressors. VSMC-KO.

File name: Supplementary Data 9
Description: IPA analysis results for disease and biofunctions (DBF) of cells exposed to all stressors. EC.

File name: Supplementary Data 10
Description: IPA analysis results for upstream regulator analysis (URA) of cells exposed to all stressors. Comparison_VSMC-WT_VSMCKO_EC.

File name: Supplementary Data 11
Description: IPA analysis results for upstream regulator analysis (URA) of cells exposed to all stressors. VSMC-WT.

File name: Supplementary Data 12
Description: IPA analysis results for upstream regulator analysis (URA) of cells exposed to all stressors. VSMC-KO.

File name: Supplementary Data 13
Description: IPA analysis results for upstream regulator analysis (URA) of cells exposed to all stressors. EC.

File name: Supplementary Data 14
Description: Results of gene ontology enrichment analysis with g:Profiler. VSMC-WT.

File name: Supplementary Data15
Description: Results of gene ontology enrichment analysis with g:Profiler. VSMC-KO.

File name: Supplementary Data 16
Description: Masterfiles with all FPM values. VSMC.

File name: Supplementary Data 17
Description: Masterfiles with all FPM values. EC.

File name: Supplementary Data 18
Description: Lists of genes for enrichment analysis. VSMCWT, ALL versus CON.

File name: Supplementary Data 19
Description: Lists of genes for enrichment analysis. VSMCWT, HG+FFA versus CON.

File name: Supplementary Data 20
Description: Lists of genes for enrichment analysis. VSMCKO, ALL versus CON.

File name: Supplementary Data 21
Description: Lists of genes for enrichment analysis. VSMCKO, HG+FFA versus CON.

File name: Supplementary Data 22
Description: Lists of genes for enrichment analysis. EC, ALL versus CON.

File name: Supplementary Data 23
Description: Lists of genes for enrichment analysis. EC, HG+FFA versus CON.

File name: Supplementary Data 24
Description: Numerical source data of the figures, unless these data are already part of supplementary data.
