# Supplementary material for: Early-phase impact of obesity-associated stress on murine vascular smooth muscle cells depends on EGFR and sex
Source: Commun Biol. 2025 Dec 22;8:1834. doi: 10.1038/s42003-025-09416-7 (PMC12749158; doi:10.1038/s42003-025-09416-7)
Supplement: Supplementary file 27 — Reporting Summary [file 42003_2025_9416_MOESM27_ESM.pdf]

nature portfolio

Corresponding author(s): Michael Gekle

Last updated by author(s): Dec 2, 2025

Reporting Summary

Nature Portfolio wishes to improve the reproducibility of the work that we publish. This form provides structure for consistency and transparency in reporting. For further information on Nature Portfolio policies, see our Editorial Policies and the Editorial Policy Checklist.

Statistics

For all statistical analyses, confirm that the following items are present in the figure legend, table legend, main text, or Methods section.

n/a	Confirmed	
☐	☒	The exact sample size (*n*) for each experimental group/condition, given as a discrete number and unit of measurement
☐	☒	A statement on whether measurements were taken from distinct samples or whether the same sample was measured repeatedly
☐	☒	The statistical test(s) used AND whether they are one- or two-sided<br>*Only common tests should be described solely by name; describe more complex techniques in the Methods section.*
☒	☐	A description of all covariates tested
☐	☒	A description of any assumptions or corrections, such as tests of normality and adjustment for multiple comparisons
☐	☒	A full description of the statistical parameters including central tendency (e.g. means) or other basic estimates (e.g. regression coefficient) AND variation (e.g. standard deviation) or associated estimates of uncertainty (e.g. confidence intervals)
☐	☒	For null hypothesis testing, the test statistic (e.g. *F*, *t*, *r*) with confidence intervals, effect sizes, degrees of freedom and *P* value noted<br>*Give P values as exact values whenever suitable.*
☒	☐	For Bayesian analysis, information on the choice of priors and Markov chain Monte Carlo settings
☒	☐	For hierarchical and complex designs, identification of the appropriate level for tests and full reporting of outcomes
☒	☐	Estimates of effect sizes (e.g. Cohen's *d*, Pearson's *r*), indicating how they were calculated

Our web collection on statistics for biologists contains articles on many of the points above.

Software and code

Policy information about availability of computer code

Data collection

Bulk RNA sequencing:
Novogene Co., Ltd (Cambridge, United-Kingdom) carried out the sequencing libraries preparation (poly(A) enrichment) and the paired-end sequencing (2 x 150 bp) runs on a NovaSeq6000 Illumina sys-tem (N = 4-10 for each condition, from 4-9 different male animals). Adaptor clipping and data quality control was provided by the service company as well. Read mapping to the mouse genome (mm39) was done with HISAT2 (v. 2.1.0) and featureCounts (2.0.0, –M –t exon) was used to count the mapped reads. Gene annotation was done using BiomaRt (v.2.60.0) to access Ensembl archive v109.
Digital microscopy:
Images were acquired by digital microscopy (10x objective, Cytation3 imaging reader, BioTek, Germany) using the Gene 5 Image Prime 3.16 software (BioTek, Bad Friedrichshall, Germany).

Data analysis

Differential expression analysis:
Differential expression analysis was performed using edgeR (4.2.0) and DESeq2 (1.44.0). Due to a major influence on the data caused by the cell type, analyses were performed independently for each of them (WT VSMC, EGFR-KO VSMC, EC). For each of these analyses, based on the multiple variables influencing overall gene expression, the design with strict sample pairing of animal + treatment was applied. Genes with sufficient counts to be considered in the statistical analyses were filtered using the filterByExpr edgeR function and the independent filtering parameter (a = 0.05) of the DESeq2 results function. In the edgeR analysis, normalization factors were calculated with the ‘‘trimmed mean of M value’’ (TMM) method, and the data fitting and testing were performed with the glmGLFit and glmQLFTest functions, respectively. Significantly ‘‘differentially expressed genes’’ (DEG) were defined as genes with a false discovery rate (FDR) below 0.05 in both DESeq2 and edgeR outputs (overlap of the respective results), with at least 3 FPM on average in one of the sample groups considered for a given comparison and with |log2 Fold Change| > 0.64 (threshold based on the inherent variation in control samples: threshold = 3 x [average coefficient of variation] from all RNAs).

Clustering of regulated protein-coding genes:
The STRING database (https://string-db.org/) and its associated tools were used to identify association networks, as well as functional clusters, among the regulated genes. For each group of genes, a full STRING network was built, with a minimum required interaction score set at 0.7 (high confidence). The edge thickness indicates the strength of the data supporting the association between two nodes, and the dotted lines represent edges between clusters. Disconnected nodes were not displayed. “K-means clustering” method served to identify clusters within the network, and enriched Gene Ontology terms and (Reactome-, KEGG-, Wiki-) pathways were automatically returned for each of them.
Gene ontology enrichment analysis:
Gene Ontology (GO) term enrichment analysis was performed with the web server g:profiler2 (https://biit.cs.ut.ee/gprofiler/orth). Single and multiple queries were performed. For each condition, a list of regulated genes served as input. Only GO terms comprising less than 5000 genes were considered and those were simultaneously filtered for an adjusted p-value below 0.05 for all datasets. The enrichment score E of the filtered GO terms was calculated, with E = (intersection size/query size) / (term size/effective domain size).
Ingenuity Pathway Analysis:
The lists of differentially expressed genes were uploaded in the Ingenuity Pathway Analysis (IPA) application (Qiagen, Germany) to perform enrichment analysis. Core analyses were performed in order to predict which molecular and biofunctions, regulatory pathways or canonical pathways (filtered to not include overrepresented cancer-related terms) may be regulated by metabolic and humoral stressors, but also to predict which factors may be involved in the regulation of these genes (i.e. upstream regulators). Based on the IPA internal database, predicted activation states were returned for each as Z-scores (positive and negative Z-scores corresponding to putative activation and inhibition, respective-ly). The outputs of these analyses were aligned with the incorporated “Comparison analysis” tool. The thresholds were set at |Z-score|≥2 and a Benjamini Hochberg (BH) p-value ≤0.01.
Digital microscopy:
Images were analyzed with the Gene 5 Image Prime 3.16 software (BioTek, Bad Friedrichshall, Germany) and in-build routines after adjusting the necessary parameters (background, threshold, object size, rolling ball size).
Statistical analysis:
Data are presented either as box plots or as mean [illegible] 95% confidence intervals. ANOVA on rank test followed by post hoc testing or Wilcoxon rank sum tests were used because pre-test data analysis by SigmaPlot 12.5 indicated that most of the data were not normally distributed. Biometrical planning was performed under consideration of the 3R-principle with α = 0.05 and β = 0.8. For cell culture experi-ments, cells from at least 4 different animals were used.
Our experimental design provided strictly connected sets of samples for control, metabolic stressors, humoral stressors and combined stressors originating from the same animal, at the same cell passage, treated at exactly the same time (suppl. figure SF1). Thus, each set was biologically independent. This design allowed us to calculate the relative effects of the stressors in a paired way (stressor effecti = valuei,stressors/valuei,control for i [illegible] [1-N], with N the final number of replicates), which greatly reduced ef-fects of passage or animal. Subsequently, we could calculate the 95% or 99% confidence intervals and test the exclusion of the value = 1 (corresponding to a difference from controls with alpha < 0.05 or 0.01, respectively). This procedure was applied in part to the analysis of gene expression as well as to the functional assays.

For manuscripts utilizing custom algorithms or software that are central to the research but not yet described in published literature, software must be made available to editors and reviewers. We strongly encourage code deposition in a community repository (e.g. GitHub). See the Nature Portfolio guidelines for submitting code & software for further information.

Data

Policy information about availability of data

All manuscripts must include a data availability statement. This statement should provide the following information, where applicable:

- Accession codes, unique identifiers, or web links for publicly available datasets
- A description of any restrictions on data availability
- For clinical datasets or third party data, please ensure that the statement adheres to our policy

The datasets generated during and/or analysed during the current study are available in the gene expression omnibus database with the study identity GSE294868. (Token: mxmpcimsldyvzmp).
Further data are provided in the supplementary tables ST01, ST02 a-d, ST03 a-d, ST04 a-d, ST05 a-b, ST06 a-b, ST07 a-f, which are part of the mansucript.

Research involving human participants, their data, or biological material

Policy information about studies with human participants or human data. See also policy information about sex, gender (identity/presentation), and sexual orientation and race, ethnicity and racism.

Reporting on sex and gender

Use the terms sex (biological attribute) and gender (shaped by social and cultural circumstances) carefully in order to avoid confusing both terms. Indicate if findings apply to only one sex or gender; describe whether sex and gender were considered in study design; whether sex and/or gender was determined based on self-reporting or assigned and methods used.
Provide in the source data disaggregated sex and gender data, where this information has been collected, and if consent has been obtained for sharing of individual-level data; provide overall numbers in this Reporting Summary. Please state if this information has not been collected.
Report sex- and gender-based analyses where performed, justify reasons for lack of sex- and gender-based analysis.

Reporting on race, ethnicity, or other socially relevant groupings

Please specify the socially constructed or socially relevant categorization variable(s) used in your manuscript and explain why they were used. Please note that such variables should not be used as proxies for other socially constructed/relevant variables (for example, race or ethnicity should not be used as a proxy for socioeconomic status).
Provide clear definitions of the relevant terms used, how they were provided (by the participants/respondents, the researchers, or third parties), and the method(s) used to classify people into the different categories (e.g. self-report, census or administrative data, social media data, etc.)
Please provide details about how you controlled for confounding variables in your analyses.

Population characteristics

Describe the covariate-relevant population characteristics of the human research participants (e.g. age, genotypic

Population characteristics	*information, past and current diagnosis and treatment categories). If you filled out the behavioural & social sciences study design questions and have nothing to add here, write "See above."*
Recruitment	*Describe how participants were recruited. Outline any potential self-selection bias or other biases that may be present and how these are likely to impact results.*
Ethics oversight	*Identify the organization(s) that approved the study protocol.*

Note that full information on the approval of the study protocol must also be provided in the manuscript.

Field-specific reporting

Please select the one below that is the best fit for your research. If you are not sure, read the appropriate sections before making your selection.

☒ Life sciences ☐ Behavioural & social sciences ☐ Ecological, evolutionary & environmental sciences

For a reference copy of the document with all sections, see nature.com/documents/nr-reporting-summary-flat.pdf

Life sciences study design

All studies must disclose on these points even when the disclosure is negative.

Sample size	For bulk RNA sequencing sapmle sets from 4-9 different male animals from each genotype were used.<br>For cell culture experiments, cells from at least 4 different animals were used.<br>Our experimental design provided strictly connected sets of samples for control, metabolic stressors, humoral stressors and combined stressors originating from the same animal, at the same cell passage, treated at exactly the same time. Thus, each set was biologically independent. This design allowed us to calculate the relative effects of the stressors in a paired way (stressor effecti = valuei,stressors/ valuei,control for i E [1-N], with N the final number of replicates), which greatly reduced effects of passage or animal. Subsequently, we could calculate the 95% or 99% confidence intervals and test the exclusion of the value = 1 (corresponding to a difference from controls with alpha < 0.05 or 0.01, respectively). This procedure was applied in part to the analysis of gene expression as well as to the functional assays.<br>One experimental set consisted of 5 to 6 wells for each of the four incubation conditions. 6 to 8 independent plates (different animals, different passages) were used for each paratmeter (i.e. 6 to 8 independent biological replicates). For statistical testing the results of 5-6 well from one plate were aggregated and the number of plates represents N during testing.
Data exclusions	Only complete experimental set with technically approved values for each of the four conditions were included.
Replication	Our experimental design provided strictly connected sets of samples for control, metabolic stressors, humoral stressors and combined stressors originating from the same animal, at the same cell passage, treated at exactly the same time. Thus, each set was biologically independent. This design allowed us to calculate the relative effects of the stressors in a paired way (stressor effecti = valuei,stressors/ valuei,control for i E [1-N], with N the final number of replicates), which greatly reduced effects of passage or animal. Subsequently, we could calculate the 95% or 99% confidence intervals and test the exclusion of the value = 1 (corresponding to a difference from controls with alpha < 0.05 or 0.01, respectively). This procedure was applied in part to the analysis of gene expression as well as to the functional assays.<br>One experimental set consisted of 5 to 6 wells for each of the four incubation conditions. 6 to 8 independent plates (different animals, different passages) were used for each paratmeter (i.e. 6 to 8 independent biological replicates). For statistical testing the results of 5-6 well from one plate were aggregated and the number of plates represents N during testing.
Randomization	For each parameter values were obtained in parallel (i.e. in a paired manner) from cells of each donor at each passage. Thus, cells of each donor at each passage were exposed to all conditions at the same time.
Blinding	Exposure of the cells and data aquisition/analysis were performed by two different persons.

Reporting for specific materials, systems and methods

We require information from authors about some types of materials, experimental systems and methods used in many studies. Here, indicate whether each material, system or method listed is relevant to your study. If you are not sure if a list item applies to your research, read the appropriate section before selecting a response.

Materials & experimental systems

n/a	Involved in the study	
☐	☒	Antibodies
☒	☐	Eukaryotic cell lines
☒	☐	Palaeontology and archaeology
☐	☒	Animals and other organisms
☒	☐	Clinical data
☒	☐	Dual use research of concern
☒	☐	Plants

Methods

n/a	Involved in the study	
☒	☐	ChIP-seq
☒	☐	Flow cytometry
☒	☐	MRI-based neuroimaging

Antibodies

Antibodies used	From Cell Signaling Technologies, Frankfurt, Germany: Anti-EGFR XP #4267, 1:2000; SRF #5147, 1:2000; phospho-SRFS103 #4261, 1:1000; ELK-1 #9182, 1:500; phospho-ELK1S383 #9186, 1:500; MRTF-A #14760, 1:1000; MRTF-B #14613, 1:1000; phospho-EGFRY1068 #3777, 1:1000; Anti-HSP90 #4874, 1:2000; Anti-Rabbit IgG HRP #7074, 1:1000.<br>From abcam, Cambridge, UK: HB-EGF #ab192545 1:1000.<br>From Becton Dickinson: Anti-BrdU antibody #347580, 1:200.<br>From Invitrogen: Red fluorescent secondary antibody #A10037, 1:250.

Validation

Anti-BrdU:
The Anti-BrdU antibody, clone B44, is derived from hybridization of Sp2/0-Ag14 mouse myeloma cells with spleen cells from BALB/c mice immunized with iodouridine-conjugated ovalbumin. Bromodeoxyuridine (BrdU) is a uridine derivative that can be incorporated specifically into DNA in place of thymidine. Anti-BrdU identifies BrdU (but not thymidine) in single-stranded DNA, free BrdU, or BrdU coupled to a protein carrier. The antibody also reacts with iodouridine.
There are several publications describing the use of this antibody in murine cells or tissue. E.g.
Chemerin regulates normal angiogenesis and hypoxia-driven neovascularization.
In Angiogenesis on 1 May 2022 by Ben Dhaou, C., Mandi, K., et al.
Functional interplay between c-Myc and Max in B lymphocyte differentiation.
In EMBO Reports on 1 October 2018 by Perez-Olivares, M., Trento, A., et al.
HDAC1 and HDAC2 integrate checkpoint kinase phosphorylation and cell fate through the phosphatase-2A subunit PR130.
In Nature Communications on 22 February 2018 by Göder, A., Emmerich, C., et al.
Maternal Sevoflurane Exposure Causes Abnormal Development of Fetal Prefrontal Cortex and Induces Cognitive Dysfunction in Offspring.
In Stem Cells International on 4 November 2017 by Song, R., Ling, X., et al.
Mllt10 knockout mouse model reveals critical role of Af10-dependent H3K79 methylation in midfacial development.
In Scientific Reports on 20 September 2017 by Ogoh, H., Yamagata, K., et al.

Anti-EGFR XP:
EGF Receptor (D38B1) XP® Rabbit mAb detects endogenous levels of total EGF receptor protein. The antibody does not cross-react with other proteins of the ErbB family. Species Reactivity: Human, Mouse, Monkey. Source / Purification: Monoclonal antibody is produced by immunizing animals with a fusion protein containing the cytoplasmic domain of human EGF receptor. Various data on the website of the supplier show the validation of the antibody. In more than 1000 publications this antibody is cited.

Anti-SRF:
SRF (D71A9) Rabbit Monoclonal Antibody recognizes endogenous levels of total SRF protein. Species Reactivity: Human, Mouse, Rat. Source / Purification: Monoclonal antibody is produced by immunizing animals with a synthetic peptide corresponding to residues surrounding Ser375 of human SRF protein. Various data on the website of the supplier show the validation of the antibody. In more than 90 publications this antibody is cited.

Anti-phospho-SRFS103:
Phospho-SRF (Ser103) Antibody detects endogenous levels of SRF only when phosphorylated at serine 103. Species Reactivity: Human, Mouse, Rat. Source / Purification: Polyclonal antibodies are produced by immunizing animals with a synthetic phosphopeptide corresponding to residues around serine 103 of human SRF. Antibodies are purified by protein A and peptide affinity chromatography. Various data on the website of the supplier show the validation of the antibody. In more than 20 publications this antibody is cited.

Anti-ELK-1:
Elk-1 Antibody detects recombinant levels of total Elk-1 protein. This antibody may cross-react with other Elk/ETS-domain family members. Species Reactivity: Human, Mouse. Source / Purification: Polyclonal antibodies are produced by immunizing animals with a synthetic peptide corresponding to the sequence of human Elk-1. Antibodies are purified by protein A and peptide affinity chromatography. Various data on the website of the supplier show the validation of the antibody. In more than 20 publications this antibody is cited.

Anti-phospho-ELK1S383:
Phospho-Elk-1 (Ser383) (2B1) Mouse Monoclonal Antibody detects less than 5 ng of phosphorylated Elk-1, and will not react with up to 1 µg of nonphosphorylated Elk-1 protein. Species Reactivity: Human, Mouse, Rat. Monoclonal antibody is produced by immunizing animals with a synthetic phosphopeptide corresponding to residues around Ser383 of human Elk-1. Various data on the website of the supplier show the validation of the antibody. In more than 30 publications this antibody is cited.

Anti-MRTF-A:
MKL1/MRTF-A Antibody recognizes endogenous levels of total MKL1/MRTF-A protein. Species Reactivity: Human, Mouse, Rat. Source / Purification: Polyclonal antibodies are produced by immunizing animals with a synthetic peptide corresponding to residues surrounding Gly812 of human MKL1/MRTF-A protein. Antibodies are purified by protein A and peptide affinity chromatography. The antibody is cited in more than 20 publications.

Anti-MRTF-B:
MKL2/MRTF-B Antibody recognizes endogenous levels of total MKL2/MRTF-B protein. Species Reactivity: Human, Mouse. Source / Purification: Polyclonal antibodies are produced by immunizing animals with a synthetic peptide corresponding to residues surrounding Gln1081 of human MKL2/MRTF-B protein. Antibodies are purified by protein A and peptide affinity chromatography. The antibody is cited in 9 publications.

Anti-phospho-EGFRY1068:
Phospho-EGF Receptor (Tyr1068) (D7A5) Rabbit Monoclonal Antibody detects endogenous EGF receptor only when phosphorylated at Tyr1068. This antibody may cross-react weakly with other tyrosine-phosphorylated proteins. Species Reactivity: Human, Mouse,

Rat, Monkey. Source / Purification: Monoclonal antibody is produced by immunizing animals with a synthetic phosphopeptide corresponding to residues surrounding Tyr1068 of human EGF receptor. Various data on the website of the supplier show the validation of the antibody. In more than 1000 publications this antibody is cited.

Anti-HB-EGF:
Rabbit polyclonal to HBEGF. This antibody was raised against recombinant full length protein corresponding to Human HBEGF/DTR. It was affinity-purified from rabbit antiserum by affinity-chromatography using epitope-specific immunogen and the purity is > 95% (by SDS-PAGE). Species reactivity: Mouse, Rat, Human. The antibody is cited in 10 publications.

Anti-HSP90:
HSP90 Antibody detects endogenous levels of total HSP90 protein, alpha and beta isoforms. This antibody does not cross-react with other HSPs. Species Reactivity: Human, Mouse, Rat, Monkey, D. melanogaster, Zebrafish. Source / Purification: Polyclonal antibodies are produced by immunizing animals with synthetic peptides corresponding to human HSP90. Antibodies are purified by protein A and peptide affinity chromatography. Various data on the website of the supplier show the validation of the antibody. In more than 500 publications this antibody is cited.

Red fluorescent secondary antibody:
Donkey anti-Mouse IgG (H+L) Highly Cross-Adsorbed Secondary Antibody, Alexa Fluor™ 568. The donkey anti-mouse IgG whole secondary antibody has been affinity-purified and show minimum cross-reactivity to bovine, chicken, goat, guinea pig, hamster, horse, human, rabbit, rat, and sheep serum proteins. Cross-adsorption or pre-adsorption is a purification step to increase specificity of the antibody resulting in higher sensitivity and less background staining. The secondary antibody solution is passed through a column matrix containing immobilized serum proteins from potentially cross-reactive species. Only the nonspecific-binding secondary antibodies are captured in the column, and the highly specific secondaries flow through. Species Reactivity: Mouse. Host/Isotype: Donkey / IgG. Class: Polyclonal. Immunogen: Gamma Immunoglobin. Conjugate: Alexa Fluor™ 568. In more than 1000 publications this antibody is cited.

Anti-Rabbit IgG HRP:
Horse radish peroxidase (HRP)-coupled secondary antibody designed for use with rabbit polyclonal and monoclonal antibodies, this affinity purified goat anti-rabbit IgG (heavy and light chain) antibody is conjugated to horseradish peroxidase(HRP) for chemiluminescent detection. Species Reactivity: Rabbit. In more than 10000 publications this antibody is cited.

Immunoblotting:
Cells were lysed with CST lysis buffer (20mM Tris, pH 7.5 (Illinois Tools Works companies), 150mM NaCl (Roth), 1% Triton X-100 (Sigma-Aldrich), 1mM EDTA (Merck), 1mM EGTA (Sigma-Aldrich), 184mg/L Na-orthovanadate (Sigma-Aldrich), 2.5mM Na-pyrophosphate (Sigma-Aldrich), 1mM β-glycerolphosphate (Sigma-Aldrich)), centrifuged at 13.000 g for 10 minutes and protein amount was determined with Bradford assay. Equal amounts of the proteins were denaturated with 6x Laemmli buffer (0.5M Tris pH 6.8 (Roth GmbH), 10% SDS (Roth), 10% Glycer-ol (Sigma-Aldrich)) at 95°C for 5-10 minutes. Proteins were separated by 10% sodium dodecyl sulfate–polyacrylamide gel electrophoresis (SDS-PAGE) and transferred onto a nitrocellulose membrane. After blocking with 5 % nonfat dry milk powder in Tris-buffered saline with Tween20 (TBS-Tween) (20mM Tris base, pH 7.4 (Illinois Tools Works companies), 150mM NaCl, 0.05% Tween-20 (Sigma-Aldrich)) membranes were incubated with first antibody diluted in 5 % bovine serum albumin (BSA) in TBS-Tween overnight. Horse radish peroxidase (HRP)-coupled secondary antibodies, 1:1000 in 5 % nonfat dry milk powder in TBS-Tween) were used. After removal of unbound secondary antibody three washing steps in TBS-TWEEN were performed. Finally Clarity™ Western ECL Substrate (Bio-Rad, Munich, Germany) was added and the peroxidase activity-based light emission was recorded by an imaging system (Image Quant LAS4000, GE Health care, Buckinghamshire, GB). The antibodies used are de-scribed below. Densitometry analysis was per-formed with Quantity One� software from BioRad (Feldkirchen, Germany).

Animals and other research organisms

Policy information about studies involving animals; ARRIVE guidelines recommended for reporting animal research, and Sex and Gender in Research

Laboratory animals	Recently, we generated and described a conditional knock out for EGFR in VSMC via the Cre/loxP sys-tem in combination with EGFRflox/flox C57BL/6J mice (originally provided by Maria Sibilia, Vienna, Austria). C57BL/6 mice containing floxed EGFR alleles (EGFR f/f) after removal of the neo cassette were used for further breeding. EGFR was inactivated tissue-specifically in vascular smooth muscle cells (VSMC) by using SM22-cre transgenic mice, in which the CRE recombinase is under the control of the VSMC-specific SM22 promoter. Mice were kept at constant temperature of 22±2°C, relative humidity of 30–60%, under a 12/12 h light-dark cycle with ad libitum access to water and standard chow.
Wild animals	*Provide details on animals observed in or captured in the field; report species and age where possible. Describe how animals were caught and transported and what happened to captive animals after the study (if killed, explain why and describe method; if released, say where and when) OR state that the study did not involve wild animals.*
Reporting on sex	RNASeq data were obtained from cells of male animals. Cell biological data were obtained from cells of male and in part of female animals.
Field-collected samples	*For laboratory work with field-collected samples, describe all relevant parameters such as housing, maintenance, temperature, photoperiod and end-of-experiment protocol OR state that the study did not involve samples collected from the field.*
Ethics oversight	All mouse experiments were approved by the local government (Landesverwaltungsamt Sachsen-Anhalt, Germany, Az.: 505.6.3-42502-2-1389 MLU_G; Veterinäramt Stadt Halle, Germany; Bescheid T16/2019) and conducted in accordance with the National Institutes of Health Guide for the Care and Use of Laboratory Animals, the ARRIVE guidelines and under consideration of the 3R-principle.

Note that full information on the approval of the study protocol must also be provided in the manuscript.

Plants

Seed stocks

Report on the source of all seed stocks or other plant material used. If applicable, state the seed stock centre and catalogue number. If plant specimens were collected from the field, describe the collection location, date and sampling procedures.

Novel plant genotypes

Describe the methods by which all novel plant genotypes were produced. This includes those generated by transgenic approaches, gene editing, chemical/radiation-based mutagenesis and hybridization. For transgenic lines, describe the transformation method, the number of independent lines analyzed and the generation upon which experiments were performed. For gene-edited lines, describe the editor used, the endogenous sequence targeted for editing, the targeting guide RNA sequence (if applicable) and how the editor was applied.

Authentication

Describe any authentication procedures for each seed stock used or novel genotype generated. Describe any experiments used to assess the effect of a mutation and, where applicable, how potential secondary effects (e.g. second site T-DNA insertions, mosiacism, off-target gene editing) were examined.
